# Novel phosphorylation states of the yeast spindle pole body

**DOI:** 10.1242/bio.033647

**Published:** 2018-06-14

**Authors:** Kimberly K. Fong, Alex Zelter, Beth Graczyk, Jill M. Hoyt, Michael Riffle, Richard Johnson, Michael J. MacCoss, Trisha N. Davis

**Affiliations:** 1 Department of Biochemistry, University of Washington, Seattle, WA 98195, USA; 2 Department of Genome Sciences, University of Washington, Seattle, WA 98195, USA

**Keywords:** Spindle pole body (SPB), Cell cycle, Phosphoproteome, γ-tubulin small complex (γ-TuSC)

## Abstract

Phosphorylation regulates yeast spindle pole body (SPB) duplication and separation and likely regulates microtubule nucleation. We report a phosphoproteomic analysis using tandem mass spectrometry of enriched *Saccharomyces cerevisiae* SPBs for two cell cycle arrests, G1/S and the mitotic checkpoint, expanding on previously reported phosphoproteomic data sets. We present a novel phosphoproteomic state of SPBs arrested in G1/S by a *cdc4-1* temperature-sensitive mutation, with particular focus on phosphorylation events on the γ-tubulin small complex (γ-TuSC). The *cdc4-1* arrest is the earliest arrest at which microtubule nucleation has occurred at the newly duplicated SPB. Several novel phosphorylation sites were identified in G1/S and during mitosis on the microtubule nucleating γ-TuSC. These sites were analyzed *in vivo* by fluorescence microscopy and were shown to be required for proper regulation of spindle length. Additionally, *in vivo* analysis of two mitotic sites in Spc97 found that phosphorylation of at least one of these sites is required for progression through the cell cycle. This phosphoproteomic data set not only broadens the scope of the phosphoproteome of SPBs, it also identifies several γ-TuSC phosphorylation sites that influence microtubule formation.

## Abstract (precis)

**Summary:** A phosphoproteome of yeast spindle pole bodies in G1/S or M phase identifies phosphorylation sites involved in spindle length control and provides direction for future phosphorylation analyses of spindle pole components.

## INTRODUCTION

The centrosome is the microtubule organizing center of the cell, responsible for nucleating microtubules and establishing a bipolar spindle during mitosis. In budding yeast, the spindle pole body (SPB) is the functional equivalent of the centrosome in higher eukaryotic cells and the essential yeast spindle pole components have homologues in humans (Flory and Davis, 2003; Jaspersen and Winey, 2004; Lin et al., 2014; Oakley et al., 1990; Stearns et al., 1991). However, the yeast SPB exhibits a morphologically distinct structure from higher eukaryotic centrosomes. In contrast to the fluid matrix of the pericentriolar material built around the centrioles of mammalian centrosomes (Woodruff et al., 2014, 2015), the SPB exists as three highly organized, stratified layers built around a crystalline core of Spc42 (Bullitt et al., 1997; Byers and Goetsch, 1975; O'Toole et al., 1999; Viswanath et al., 2017). Our understanding of SPB structure and composition has been advantageously used to understand highly conserved mechanisms of centrosomal regulation through phosphorylation (Bouhlel et al., 2015; Jaspersen et al., 2004; Winey et al., 1991). Centrosomes serve as signaling platforms, integrating cell signals to regulate the localization of spindle proteins and progression through the cell cycle (Basto and Pines, 2007; Burns et al., 2015; Casenghi et al., 2005; Cha et al., 2004; Jaspersen and Winey, 2004; Jiang et al., 2006; Lange, 2002; Rieder et al., 2001; Winey and Bloom, 2012). More specifically, kinases Mps1, Polo, Hrr25, and Cdk1 have been implicated in the regulation of SPB duplication, SPB separation, and cell cycle transitions (Avena et al., 2014; Castillo et al., 2002; Deshaies and Ferrell, 2001; Jaspersen and Winey, 2004; Jaspersen et al., 2004; Peng et al., 2015; Winey et al., 1991).

Previous research shows that all SPB proteins are phosphoproteins. Two of the best characterized phosphoproteins at the SPB are Tub4 and Spc110, both implicated in microtubule nucleation. Several individual phosphorylation sites have been identified and mutated in Tub4. Phosphomimetic mutations of highly conserved Tub4 sites induce a mitotic arrest and confer defects in spindle assembly (S360) (Keck et al., 2011; Lin et al., 2011), increase microtubule assembly rates and numbers of microtubules at the SPB (Y445) (Vogel et al., 2001), and induce metaphase arrests with short, disorganized spindles (S74 and S100) (Lin et al., 2011). Likewise, Spc110 phosphorylation has been studied extensively *in vivo* (Friedman et al., 1996, 2001; Huisman et al., 2007; Stirling and Stark, 1996). Mps1 phosphorylation of Spc110 S60, T64, and T68 is responsible for a gel shift during mitosis (Friedman et al., 1996, 2001; Stirling and Stark, 1996) and blocking phosphorylation of S91 induces a metaphase delay (Huisman et al., 2007). A compilation of previously studied spindle pole component phosphorylation sites can be found in the Supplementary Data S1.

Two large-scale analyses of SPB phosphorylation have been performed. Valuable identification of phosphorylation sites in the γ-tubulin small complex (γ-TuSC) relied on material overexpressed in yeast, which could alter the phosphorylation pattern (Lin et al., 2011). Phosphoanalysis of enriched SPBs identified 298 sites but sequence coverage of the γ-TuSC was poor compared to the coverage of the other components (Keck et al., 2011).

Using our improved SPB enrichment protocol (Fong et al., 2016) and relying on advances in mass spectrometers and analysis packages, we have conducted a new phosphoanalysis of isolated SPBs. Our data expand on previously reported phosphoproteomic data sets and identify novel cell cycle phosphorylation states of the SPB at the onset of microtubule nucleation in G1/S, a cell cycle stage not previously examined. Our phosphorylation data of the microtubule nucleating γ-TuSC builds on the current literature by identifying and fluorescently analyzing new cell cycle phosphorylation sites of the specific population of γ-TuSCs attached to SPBs.

## RESULTS AND DISCUSSION

### Enrichment by TAP-tagged Spc97 increases the yield of intact spindle pole bodies

Previous studies have shown that SPBs from *Saccharomyces cerevisiae* co-purify with Mlp2, a nuclear pore component (Niepel et al., 2005). To increase the yield of isolated SPBs, tandem affinity purification (TAP) tags were introduced on spindle pole components (List of strains in Table 1). Starting with the proteins found in the core of the SPB, we tagged Spc42 and Cnm67. While N-terminally tagged Cnm67 was viable, we found that the core spindle pole component Spc42 tagged with a C-terminal TAP tag was not viable in our strain background. In addition to core proteins, we tagged membrane anchor proteins Nbp1 and Bbp1, linker proteins Spc110 and Spc72, and γ-TuSC component Spc97 with C-terminal TAP tags.

**Table 1.**
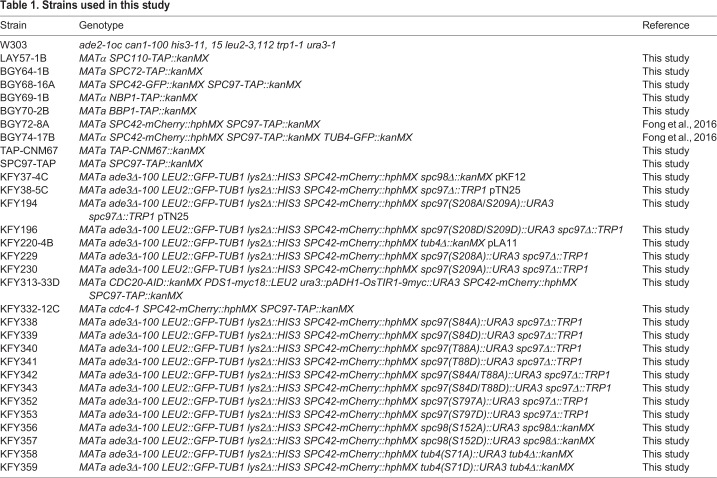
**Strains used in this study**

**Fig. 1.**
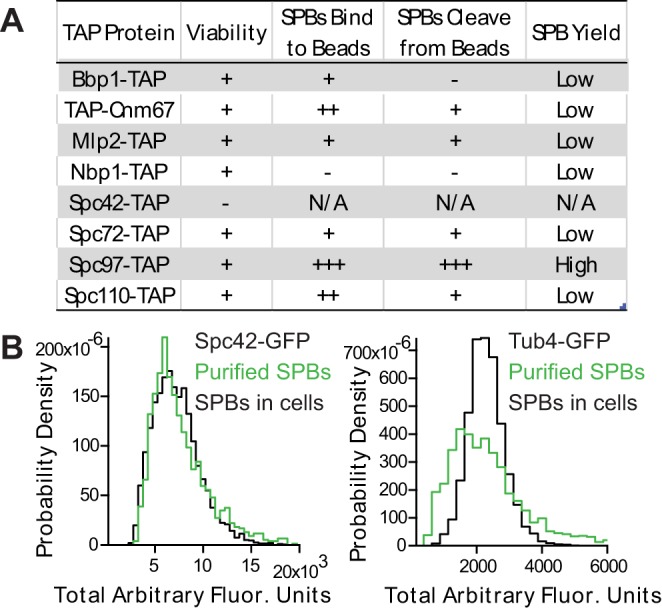
**Enrichment protocol yields intact spindle pole bodies.** (A) Several components of the spindle pole body (SPB) were tagged with a TAP tag. SPBs were isolated using each tag and the yield of SPBs determined. The efficiency of SPBs binding to beads and being cleaved from beads were determined by western blot analysis. (B) Enriched SPBs are the same size as SPBs in cells, as determined by fluorescence intensities of SPBs containing either Spc42-GFP or Tub4-GFP imaged in cells (in black) and after enrichment (in green).

We used western blot analysis to assess the yield of enriched SPBs and found that Mlp2-TAP bound weakly to IgG beads, resulting in a low yield of enriched SPBs. Similarly, Bbp1-TAP, Nbp1-TAP and Spc72-TAP showed weak or no binding to IgG beads. Other constructs (TAP-Cnm67 and Spc110-TAP) showed relatively strong binding to IgG beads, but TEV (tobacco etch virus) protease cleavage failed to remove the bound SPBs from the beads, again resulting in low yields. A C-terminal TAP tag on Spc97 was shown not only to bind the strongest to IgG beads, but to be efficiently cleaved from the beads by TEV protease, resulting in the highest, most reproducible yield of enriched SPBs (Fig. 1A).

Spc97 is a component of the γ-TuSC, a stable heterotetrameric complex that exists free in solution as well as bound to the SPB. Because our enrichment protocol relied on a TAP tag on Spc97, our protocol yielded free γ-TuSCs in addition to intact SPBs. Separation of intact SPBs and γ-TuSCs was achieved by velocity sedimentation (Fong et al., 2016). Analysis by western blot confirmed that SPBs were concentrated in fractions 9–11 of the sucrose gradient (40–50% sucrose) while the soluble fraction of the γ-TuSCs remained higher in the gradient (Fong et al., 2016).

### Enriched spindle pole bodies are the same size as spindle pole bodies *in vivo*

To determine if enriched SPBs were intact and the same size as *in vivo*, SPBs were isolated from strains that contained either Spc42-GFP or Tub4-GFP. Quantification of the fluorescence intensities verified enriched SPBs containing Spc42-GFP had the same fluorescence intensity as Spc42-GFP SPBs in live cells, suggesting that the core of the SPB remained intact through the enrichment protocol. To test if the inner and outer plaques were retained during the enrichment protocol, SPBs containing Tub4-GFP were isolated and imaged *in vitro* and *in vivo*. Quantification of fluorescence intensity indicated that isolated SPBs retained 88% of the γ-TuSC compared to SPBS in live cells (Fig. 1B). Finally, all 18 proteins were present as determined by high protein coverage in mass spectrometry (Table 2).

**Table 2.**
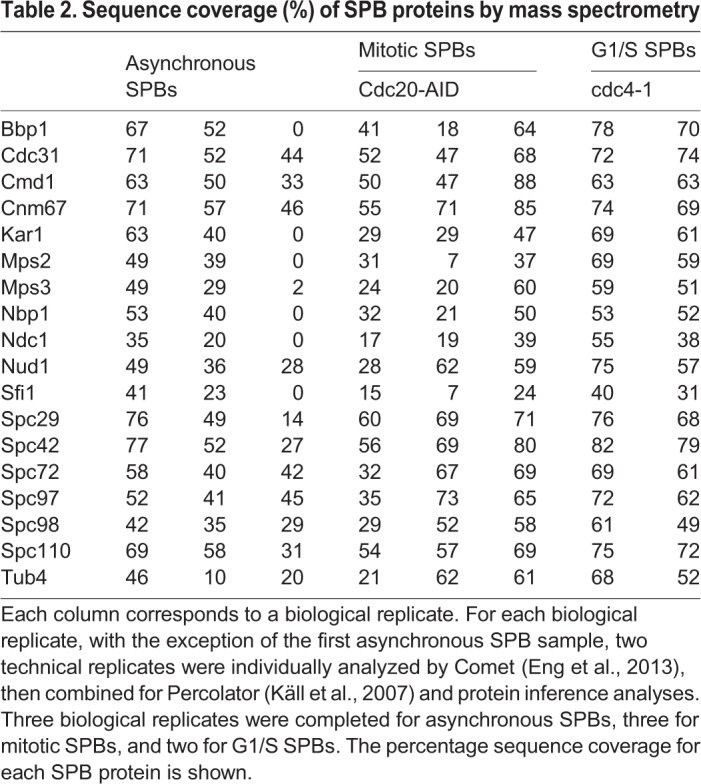
**Sequence coverage (%) of SPB proteins by mass spectrometry**

### Tandem mass spectrometry data identified phosphorylation sites of the spindle pole body at different cell cycle stages

**Fig. 2.**
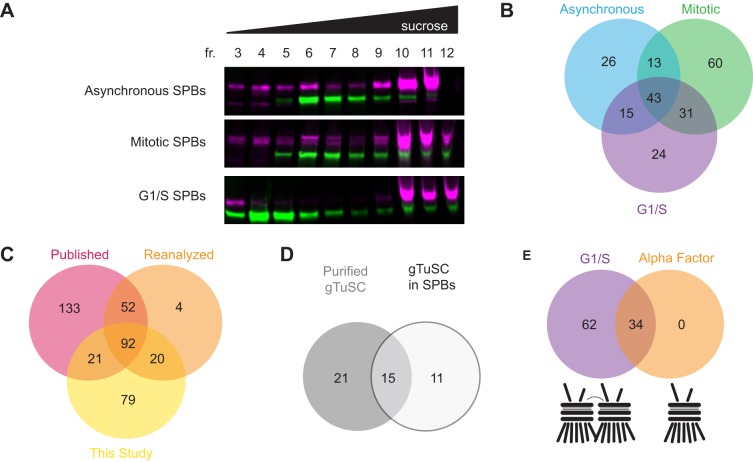
**Comparison of phosphoproteomic data sets for the spindle pole body isolated from different cell cycle stages.** (A) Asynchronous, mitotic, and G1/S SPBs were isolated. Fractions containing intact SPBs were determined by western blot analysis, using antibodies against Spc110 (in magenta) and Spc97 (in green). Sucrose concentration increases from left to right. Fractions with high concentrations of spindle pole bodies (Fractions 9, 10, 11 for asynchronous SPBs; Fractions 10, 11, for mitotic SPBs and G1/S SPBs) were used for phosphoproteomic mass spectrometry analysis. (B) Comparison of high resolution phosphoproteomic data collected in this study for asynchronous wild-type (in blue), mitotic (in green), and G1/S (in purple) SPBs. (C) Comparison of the phosphoproteome published in Keck et al. (2011) (in red), reanalysis of Keck et al. (2011) data (in orange), and data collected in this study (in yellow). (D) Comparison of γ-tubulin small complex phosphorylation sites identified in overexpressed γ-tubulin subcomplexes [Lin et al. (2011); in grey] and identified in the context of the intact SPB (this study; in white). (E) Comparison of G1 SPBs arrested by *cdc4-1* or alpha factor. In *cdc4-1* arrest, as indicated by the cartoon, the SPBs are duplicated, but not separated and both the new and the old SPBs are nucleating microtubules. In an alpha factor arrest, the SPB has not yet duplicated.

SPBs were enriched from different stages of the cell cycle to identify cell cycle specific phosphorylation events. The phosphorylation state of SPBs at G1/S had not been previously described. We isolated SPBs from *cdc4-1* cells, which were arrested in G1/S by a shift to the restrictive temperature of 36°C. To identify the subset of phosphorylation sites present during mitosis, SPBs were harvested from cells arrested in mitosis by depletion of Cdc20. Finally, we isolated SPBs from asynchronous cultures. Western blot analysis after velocity sedimentation verified the presence of SPBs in fractions 9–11 of the sucrose gradient (40–50% sucrose) using antibodies against Spc110 and Spc97 (Fig. 2A).

Phosphorylation sites were identified by mass spectrometry and verified manually to eliminate any ambiguous or unsubstantiated assignments. For the phosphoproteomic data set reported here, 212 phosphorylation sites were reported across asynchronous, mitotic, and G1/S SPBs (Fig. 2B; Supplementary Data S1). Of the sites reported in this study, 60 (28%) were only identified in mitotic SPBs, and 24 (11%) were only identified in G1/S SPBs and 26 (12%) were only detected in the asynchronous SPB sample. Forty-three sites (20%) were found in SPBs from all three conditions.

A previous study of the yeast SPB phosphoproteome reported 298 phosphorylation sites in the 18 SPB components throughout the cell cycle (Keck et al., 2011). However, since publication, advances in the resolution and sensitivity of mass spectrometers and new software packages that apply stringent quality cutoffs increased the confidence in phosphorylation assignments. Of the phosphorylation sites identified in this current study, 113 phosphorylation sites were also identified in Keck et al. (2011), resulting in 28% (113/397 total sites) of all detected sites appearing in both data sets. To determine if the difference between the data sets was due to how the mass spectra were analyzed, we reanalyzed the raw data from Keck et al. (2011) using Comet and Percolator, the same algorithms used for our new data set. The reanalysis of the Keck et al. (2011) raw data set resulted in a reduction from 298 published phosphorylation sites to 168 phosphorylation sites [for more about statistical analysis, phosphorylation assignments, reanalysis of Keck et al. (2011) data, and dataset comparisons, see Table S1 and Figs S1–S12]. Many ambiguous assignments were removed from the previously published data set by imposing strict statistical cutoffs for spectra included in the data set. The reanalysis of previously published data and our new data set showed 42% (112/268 total sites) agreement (Fig. 2C, Supplementary Data S1). The 56 sites identified in the reanalyzed published data, but not this data set, most likely result from the fact that the Keck et al. (2011) sample was enriched for phosphopeptides by running the sample over a titanium dioxide column (Keck et al., 2011). In contrast, phosphopeptides were not enriched for this data set, thus some rare phosphorylation events were likely missed. The 100 sites only identified in this study might result from improved SPB enrichment protocols or advances in mass spectrometer instrumentation and sensitivity.

Previous work focused on phosphorylation of the γ-TuSC overexpressed and purified from yeast (Lin et al., 2011). Looking at the γ-TuSC phosphorylation sites in Spc97, Spc98, and Tub4 identified in our data set, we find that 15 of the sites agreed with the reported literature. However, our data has identified 11 additional sites, six of which were identified in G1/S SPBs, on γ-TuSC that might inform our understanding of the phosphorylation state specifically when attached to the SPB (Fig. 2D).

### The phosphorylation profile of G1/S spindle pole bodies include sites present at the initiation of microtubule nucleation

Previous studies on SPB phosphorylation used alpha factor to arrest the cells in G1 (Keck et al., 2011). We report a novel phosphorylation state of SPBs from cells arrested in G1/S with a *cdc4-1* temperature sensitive arrest. These two G1 arrests differ in the state of the SPB and in the active cyclins present. In an alpha factor arrest, the SPBs are not duplicated and Cln-Cdk1 is inhibited (Gartner et al., 1998; Jeoung et al., 1998; Peter and Herskowitz, 1994). In contrast, in a *cdc4-1* temperature sensitive arrest, the SPBs are duplicated but not separated (Byers and Goetsch, 1974; Pereira et al., 1998) and there are high levels of Cln-Cdk1 and low activity of Clb-Cdk1 (Blondel et al., 2000; Duncker et al., 1999). The *cdc4-1* shift to the restrictive temperature results in the earliest cell cycle arrest in which the newly duplicated SPB has nucleated microtubules, suggesting that the phosphorylation profile at this arrest is the earliest state conducive to high microtubule nucleation activity. Interestingly, all phosphorylation events detected during an alpha factor arrest were also identified in a G1/S arrest. However, there were several additional G1/S phosphorylation events that were detected in SPBs at this stage of the cell cycle (Fig. 2E).

In alpha-factor arrested SPBs from Keck et al. (2011), very few phosphorylation sites were identified on the γ-TuSC (Spc97, Spc98 and Tub4) or the proteins that bind the γ-TuSC to the core of the SPB (Spc72 and Spc110) (Keck et al., 2011). In fact, reanalysis of the published data failed to identify any unique G1 phosphorylation events for any of these five proteins. In contrast, in this study each of the three γ-TuSC proteins – Spc97, Spc98, and Tub4 – contained phosphorylation sites only observed in G1/S SPBs as well as five G1/S phosphorylation sites on Spc110 (Supplementary Data S1).

### Phosphorylation of the γ-tubulin small complex is required for establishment of a proper mitotic spindle length

**Fig. 3.**
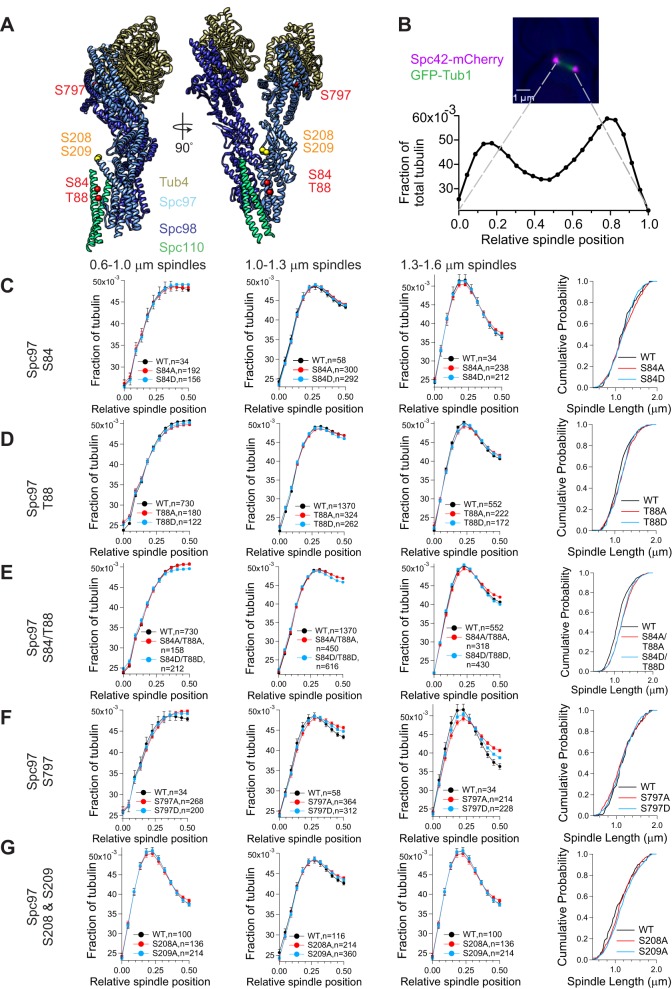
**Phosphorylation of Spc97 is required for normal mitotic spindle morphology.** (A) Mitotic and G1/S phosphorylation sites (in red) have been mapped onto the pseudo-atomic structure of Spc97 (light blue) in the yeast γ-tubulin small complex, adapted from PDB:5FM1 (Greenberg et al., 2016). Phosphoblocking mutations of Spc97 S208 and S209 (in yellow) in combination are lethal. (B) Spindle morphology was determined by measuring spindle length and tubulin fluorescence distribution along the spindle. Metaphase spindles were identified as spindles with 1.3–1.6 μm between Spc42-mCherry spindle poles. GFP-Tub1 fluorescence was measured across the entire spindle. (C–G) Half-spindle tubulin distribution profiles for spindles of a given length are shown for several Spc97 phosphorylation mutants. Spindle tubulin distributions were separated into two half spindles for ease of display (mean±s.e.m.). In each of the tubulin distribution graphs, the SPB is located at 0 and the spindle equator is located at 0.5. The far right panels show a cumulative probability of spindle length for the observed spindles, measured as the distance between Spc42-mCherry foci. Note that the x-axis begins at 0.45 μm to more clearly display the differences between traces.

We examined the role of γ-TuSC phosphorylation events on spindle morphology, focusing on sites predicted to interfere with interaction with Spc110. Spc97 S84 was identified in mitotic and G1/S SPBs and T88 was identified as a phosphorylation event in G1/S. Both of these sites were identified in free γ-TuSC, but not in previous phospho-analyses of intact SPBs (Keck et al., 2011; Lin et al., 2011). These two sites map to the outer face of γ-TuSC, in a region predicted to interact with Spc110 (Fig. 3A). Phosphomimetic and phosphoblocking mutations of these sites were integrated into yeast strains with Spc42-mCherry to label the SPBs and GFP-Tub1 to label the microtubules (list of strains in Table 1; list of plasmids in Table 3). Spindle morphology and cell growth were determined by several metrics. The distribution of tubulin fluorescence was measured across spindles of varying lengths. In wild-type cells, the tubulin fluorescence clusters in a distinct peak on either side of the spindle midzone, correlating with the kinetochore microtubules. A wider distribution of tubulin fluorescence across the half-spindle and an increase of the fraction of tubulin fluorescence at the spindle midzone indicates misregulation of microtubule length distribution (Gardner et al., 2008). Spc97 S84A, Spc97 S84D, Spc97 T88A, and Spc97 T88D had little effect on tubulin fluorescence at the spindle midzone individually or in combinations, Spc97 S84A/T88A and Spc97 S84D/T88D (Fig. 3C–E, Table 4). Another determinant of spindle morphology is the distribution of spindle lengths. The mean spindle length ±s.d. was determined for each mutation using bootstrap analysis, then compared to the mean of wild-type cells (see the Materials and Methods section). Spc97 S84A and Spc97 S84D had little effect on the distribution of spindle lengths. Mutation of Spc97 T88, either phosphoblocking or phosphomimicking, showed a statistically significant increase in spindle length distribution. The alleles carrying two mutations (S84A/T88A and S84D/T88D) both showed a further increase in spindle length (Fig. 3C–E, Table 4). And finally, cell growth was measured by budding index and comparison of large budded cell percentages. The individual mutations had little effect on the percent of large budded cells, but S84A/T88A and S84D/T88D showed a slight increase in large budded cells, suggesting a delay in the cell cycle.

**Table 3.**
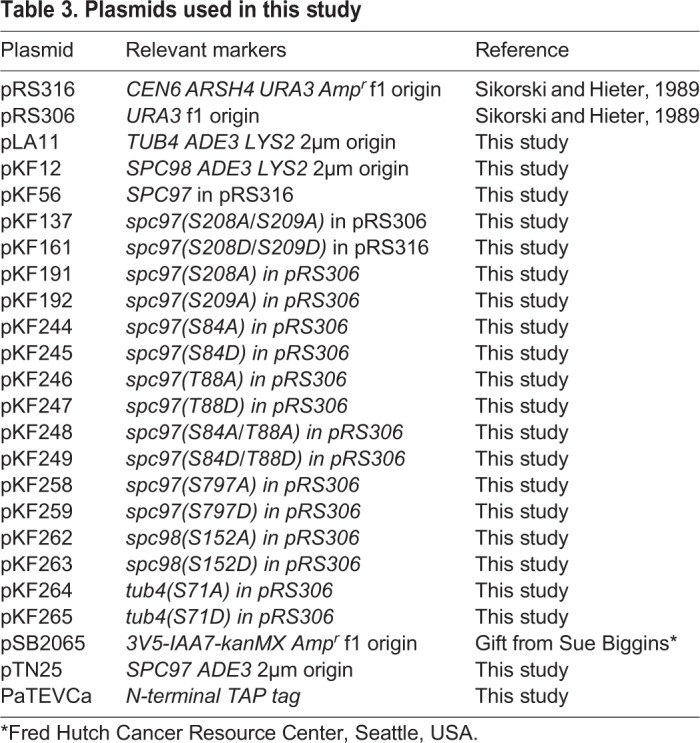
**Plasmids used in this study**

**Table 4.**
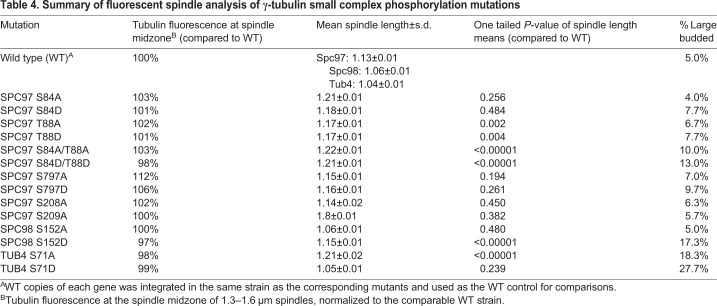
**Summary of fluorescent spindle analysis of γ-tubulin small complex phosphorylation mutations**

Similar analysis was conducted for Spc97 S797. Spc97 S797 was a novel phosphorylation site identified in mitotic SPBs and maps to the C-terminal outer face of the γ-TuSC, which could also interact with Spc110 (Fig. 3A). While phosphomimicking and phosphoblocking mutations had no effect on spindle length, tubulin distribution was affected by these mutations, with an increase of tubulin at the spindle midzone, suggestive of misregulation of kinetochore microtubule length (Fig. 3F, Table 4).

**Fig. 4.**
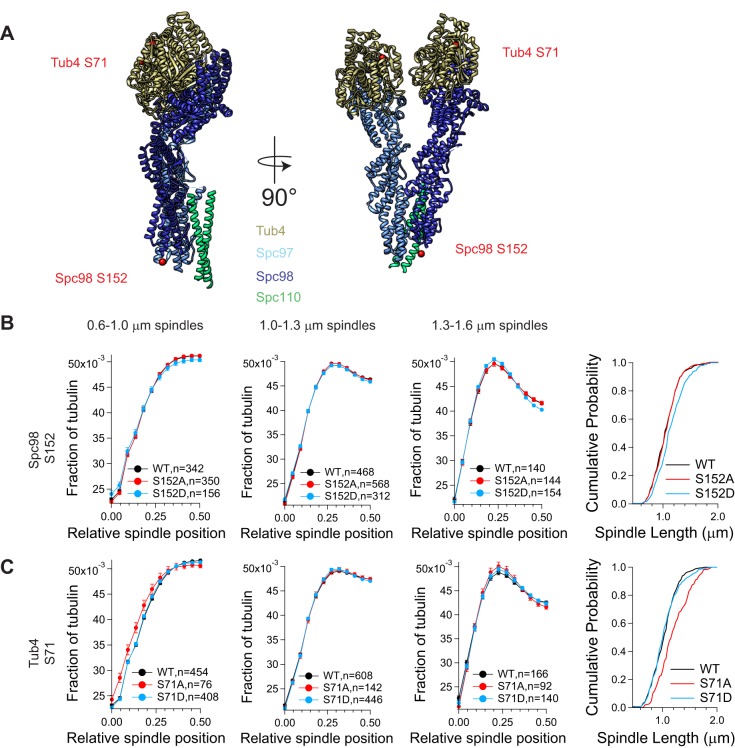
**Phosphorylation regulation of the γ-tubulin small complex is required for normal mitotic spindle morphology.** (A) Phosphorylation site S71 is shown on the pseudo-atomic structure of Tub4 (gold) in the yeast γ-tubulin small complex, adapted from PDB:5FM1 (Greenberg et al., 2016). The N-terminal 160 residues of Spc98 are not included in the pseudo-atomic structure, so the exact position of S152 is not shown. (B) Spindle morphology was determined by measuring spindle length and tubulin fluorescence distribution along the spindle. Metaphase spindles were identified as spindles with 1.3–1.6 μm between Spc42-mCherry spindle poles. GFP-Tub1 fluorescence was measured across the entire spindle. (C,D) Half-spindle tubulin distribution profiles for spindles of a given length are shown for Spc98 and Tub4 phosphorylation mutants. Spindle tubulin distributions were separated into two half spindles for ease of display (mean±s.e.m.). In each of the tubulin distribution graphs, the SPB is located at 0 and the spindle equator is located at 0.5. The far right panels show a cumulative probability of spindle length for the observed spindles, measured as the distance between Spc42-mCherry foci. Note that the x-axis begins at 0.45 μm to more clearly display the differences between traces.

Fluorescent spindle analysis was also performed for a G1/S site detected in Spc98 (S152) and a G1/S site detected in Tub4 (S71) (list of strains in Table 1; list of plasmids in Table 3). Spc98 S152 is a possible Cdk1 phosphorylation site, which was also observed in free γ-TuSC (Lin et al., 2011). While fluorescence analysis revealed no change in tubulin distribution across spindles of any length, the phosphomimetic mutation S152D showed a cell cycle delay with a statistically significant increase in the length of arrested spindles and an increase in large budded cells from 5.0% to 17.3% suggesting a checkpoint delay (Fig. 4C, Table 4). The novel G1/S phosphorylation site in Tub4 S71 sits at the interface between γ-tubulin and α-tubulin (Fig. 4A). The phosphoblocking mutation S71A resulted in longer spindles at the arrest and a cell cycle delay, with 18.3% large budded cells compared to 5.0% in wild-type cells (Fig. 4D). Phosphomimicking mutation of S71 also showed a cell cycle delay, with 27.7% large budded cells compared to 5.0% in wild-type cells; however, there was no change in spindle length distributions (Table 4).

We also examined sites identified in mitotic SPBs, hypothesizing that these sites could be involved in regulation of microtubules during the establishment of the mitotic spindle. In previously published phosphoproteomic data, γ-TuSC component Spc97 was found to have two phosphorylation sites unique to mitosis, S208 and S209 (Keck et al., 2011). Phosphomimetic mutations and phosphoblocking mutations were made for both mitotic phosphorylation sites and transformed into yeast (list of strains in Table 1; list of plasmids in Table 3). Using a plasmid shuffle assay, we determined that phosphomimicking mutations in Spc97 (S208D/S209D) were viable. However, the phosphoblocking mutations in Spc97 (S208A/S209A) were lethal. Spc97 S208A or Spc97 S209A were viable individually, with normal growth rates, morphologically wild-type spindles and wild-type tubulin distributions across metaphase spindles, suggesting that phosphorylation of at least one of these sites is required for progression through the cell cycle (Fig. 3G, Table 4). The locations of the Spc97 mitotic phosphorylation sites suggest that this region might be involved in interactions with Spc110. Disruption of the interface between Spc97 and Spc110 might destabilize the interaction between the γ-tubulin ring and Spc110.

### Conclusions

In summary, we optimized enrichment protocols to yield intact SPBs in quantities that facilitated *in vitro* analysis of SPB phosphorylation states by high-resolution mass spectrometry. While expanding on previously reported phosphorylation data sets, we were specifically interested in identifying phosphorylation sites in the γ-TuSC present at the SPB during G1/S arrest. We posit that that these G1/S sites reveal phospho-regulatory events important for the initiation of microtubule nucleation at the newly duplicated SPB. *In vivo*, mutation of G1/S and mitotic phosphorylation sites in the γ-TuSC illustrated the sensitivity of spindle organization and length regulation to perturbations at microtubule nucleation sites. This phosphoproteome provides a data set that will guide future work studying the regulation of the SPB and provide insight into the study of individual SPB components.

## MATERIALS AND METHODS

### Strains, plasmids and media

The yeast strains used in this study were all derived from W303 and are listed in Table 1. C-terminally TAP-tagged proteins were created by PCR amplifying the *TAP*-*kanMX* cassette from the plasmid TAP-2xPA using primers that shared homology with the flanking sequences of the stop codon in the gene of interest. Cnm67 was N-terminally TAP tagged by PCR amplification of the *TAP*-*kanMX* cassette from the plasmid PaTEVCa, using primers that shared homology with the flanking DNA of the start codon of *CNM67*. C-terminal mCherry and GFP protein fusions were created by PCR amplification of the *mCherry*-*hphMX3* and the *GFP*-*kanMX* cassettes from pBS35 and pFA6-GFP(S65T)::kanMX plasmids, respectively (gifts from the Yeast Resource Center, University of Washington, Seattle, USA). The cassettes for the fluorescent proteins shared homology with the flanking sequence before the stop codon of the genes of interest. For the Cdc20-AID strain, the auxin degron IAA7 was PCR amplified from pSB2065 (gift from Sue Biggins, Fred Hutchinson Cancer Research Center, Seattle, USA) with primers that shared homology with the flanking DNA of the stop codon of the gene. The above cassettes were integrated into a diploid strain, KGY315, and verified by PCR.

The plasmids used in this study are listed in Table 2. QuikChange Lightning Multi Site-Directed Mutagenesis (Stratagene, San Diego, USA) was used to construct plasmids containing point mutations. Plasmids carrying mutations in *SPC97* were integrated at the *SPC97* locus in strain KFY38-5C (*spc97*Δ), plasmids carrying mutations in *SPC98* were integrated at the *SPC98* locus in KFY37-4C (*spc98*Δ), and plasmids carrying mutations in *TUB4* were integrated at the *TUB4* locus in KFY220-1A (*tub4*Δ) using a plasmid shuffle (Widlund and Davis, 2005). These three strains all contained GFP-Tub1 for imaging of microtubules and Spc42-mCherry to visualize spindle pole bodies.

YPD media is as described (Burke et al., 2000). SD-ura low ade and SD-lys were previously described (Sundberg et al., 1996; Tien et al., 2013).

### TAP purification and velocity sedimentation

Spindle pole bodies were isolated using a TAP-tag on Spc97, as previously described (Fong et al., 2016). Sucrose gradients were generated by allowing five steps of sucrose solutions (200 µl each of 10%, 20%, 30%, 40%, 2.5 M sucrose) to equilibrate at 4°C for 2 h. For fluorescence analysis, the sucrose solutions were made in 10 mM Bis-Tris, pH 6.5, 0.1 mM MgCl_2_. For mass spectrometry analysis, the sucrose solutions were made in 40 mM HEPES, pH 7.4, 150 mM NaCl. The TEV eluate was then applied to the sucrose gradient and spun at 50,000 rpm for 5 h at 4°C in a TLS55 rotor (Beckman Coulter, Brea, USA). SPBs isolated from *cdc4-1* cells were spun at 50,000 rpm for 4 or 4.5 h to prevent side-by-side SPBs from settling to the bottom of the sucrose gradient. Fractions (90 µl) were removed from the top of the gradient with wide-bore tips. The presence of SPBs was determined by western blot analysis, probing for Spc110 (1:2000) and Spc97 (1:3000), using antibodies raised in rabbit and chicken, respectively. Western blot analysis showed the separation of intact SPBs (fractions 9–11) from the soluble pool of SPB components (fractions 1–8).

For isolation of SPBs from cells arrested by depletion of Cdc20, a strain carrying the Cdc20-AID was grown to 80 Klett units in YPD. Auxin (indole-3-acetic acid; IAA) in DMSO was added to a final concentration of 1 mM. Cdc20 was depleted for 1.5 generations before cells were harvested. In Cdc20 depleted cells, ≥98% of cells arrested with large buds. For isolation of SPBs from cells arrested at G1/S, a strain carrying *cdc4-1* was grown at 25°C to 60 Klett units, then shifted to the restrictive temperature of 36°C for two generations before harvesting cells. 95% of cells had elongated buds and 5% had large buds as expected for a *cdc4-1* arrest. Spindle pole bodies were then enriched as previously described (Fong et al., 2016).

### Fluorescence microscopy

All images were acquired using a DeltaVision system (Applied Precision, Issaquah, USA) with an IX70 inverted microscope (Olympus, Tokyo, Japan), a U Plan Apo 100× objective (1.35 NA) and a CoolSnap HQ digital camera (Photometrics, Tuscon, USA). Exposures were 0.4 s for mCherry and GFP. Images were processed as previously described (Shimogawa et al., 2009) using custom Matlab programs (Fluorcal and Calcmate) to identify GFP and mCherry foci and quantify the fluorescence intensities. Fluorcal and Calcmate are available upon request. For live-cell imaging, cells were mounted on an agarose pad as previously described (Muller et al., 2005). Metaphase spindles were identified as spindles with 20–25 pixels (1.3–1.6 μm) between spindle poles. The GFP-tubulin fluorescence intensity distribution was measured and graphed along a normalized spindle length. Fluorescence intensity at the spindle midzone (spindle length of 0.5) was compared to wild-type values. Statistical analysis of spindle length was performed using pairwise z-tests. *P*-values were computed from pairwise z-scores: z=(μ_1_−μ_2_)/(δ_1_^2^+δ_2_^2^)^0.5^, where μ_1_ and μ_2_ are bootstrap average means spindle lengths and δ_1_^2^ and δ_2_^2^ are the corresponding standard deviations (Table 4). The budding index of strains was measured by counting 300 cells; large budded cells were defined as those with buds that were two-thirds to equal the size of the mother cell.

For *in vitro* SPB imaging, a flow cell was constructed with KOH cleaned glass coverslips. SPBs were diluted with 5X BRB80/BSA (400 mM K-PIPES, pH 6.9, 5 mM MgCl_2_, 5 mM EGTA, 40 mg ml^−1^ BSA) and KCl to a final concentration of BRB80, 8 mg ml^−1^ BSA and 500 mM KCl. The diluted SPBs were flowed into the flow cell and allowed to nonspecifically adhere to the coverslip for 30 min before imaging.

### Mass spectrometry sample preparation and digestion

Enriched SPB fractions in 40–50% sucrose, 40 mM HEPES, pH 7.4, and 150 mM NaCl, were combined such that samples were approximately 2–30 µg total protein in 0.5 to 1.5 ml of buffer plus approximately 45% sucrose. Samples were diluted 1:1 using 25 mM ammonium bicarbonate (ABC) and concentrated down to 30 µl using Amicon^®^ Ultra 0.5 ml Centrifugal Filters with a 10,000 NMWL (Millipore) according to the manufacturer's instructions. 500 µl of 25 mM ammonium bicarbonate was added to the top of the filter unit and spun through. This was repeated a total of three times. Sample volume was made up to 100 µl of 25 mM ABC and reduced in the filter unit with 10 mM dithiothreitol (DTT) at 37°C for 30 min followed by a 30 min alkylation at room temperature with 15 mM iodoacetamide. 1 µl of 0.8 µg µl^−1^ Sequencing Grade Modified Trypsin (Promega Corporation, Madison, USA) was used to digest the samples in the filter units at 37°C for 4 h at 1200 rpm in an Eppendorf Thermomixer. After digestion, peptides were spun through the filter units into a new Amicon Eppendorf tube. 100 µl of 25 mM ABC was added to the top of the filter unit and spun through into the same tube. The remaining digested sample was transferred from the filter unit into the Eppendorf tube by pipette. The digested sample was reduced to about 50 µl in a Speedvac. Sample pH was adjusted to 2 with 5 M HCl prior to storage at −80°C until mass spectrometry analysis.

### Mass spectrometry

Mass spectrometry was performed on either a Q-Exactive or Q-Exactive HF (Thermo Fisher Scientific). 3 μl of sample digest was loaded by autosampler onto a 150-μm Kasil fritted trap packed with Jupiter C12 90 Å material (Phenomenex, Torrance, USA) to a bed length of 2 cm at a flow rate of 2 μl min^−1^. After loading and desalting using a total volume of 8 μl of 0.1% formic acid plus 2% acetonitrile, the trap was brought online with either a pulled fused-silica capillary tip (75-μm i.d.) or an empty Pico-Frit column (New Objective, Woburn, USA) that was self-packed with 30 cm of Reprosil-Pur C18-AQ (3-μm bead diameter, Dr Maisch) mounted in an in-house constructed microspray source and placed in line with a Waters Nanoacquity binary UPLC pump plus autosampler. Peptides were eluted off the column using a gradient of 2–35% acetonitrile in 0.1% formic acid over 120 min, followed by 35–60% acetonitrile over 10 min at a flow rate of 250 nl min^−1^.

The Q-Exactive mass spectrometer was operated using data dependent acquisition (DDA) where a maximum of twenty MS/MS spectra were acquired per MS spectrum (scan range of m/z 400–1600). The resolution for MS and MS/MS was 60,000 and 15,000, respectively, at m/z 200. The automatic gain control (AGC) targets for MS and MS/MS were set to 3e6 and 1e5, respectively, and the maximum fill times were 50 and 25 ms, respectively. The MS/MS spectra were acquired using an isolation width of 1.6 m/z and a normalized collision energy (NCE) of 27. The underfill ratio was set to 10% and the intensity threshold set to 4e5. MS/MS acquisitions were prevented for unassigned, +1, +6 and greater precursor charge states. Dynamic exclusion (including all isotope peaks) was set for 5 or 10 s. The Q-Exactive HF was operated similarly.

### Analysis of mass spectrometry data

Mass spectra from this study and from previously published data were converted into mzML using MSconvert from ProteoWizard (Chambers et al., 2012). With the exception of one asynchronous sample, two technical replicates were run for each biological sample. For each technical replicate, proteins were identified by searching high-resolution MS/MS spectra against the SGD protein sequence database using Comet (Eng et al., 2013). A variable modification of 79.966331 on S, T or Y was included in the search to identify phosphopeptides. Peptide identifications for the two technical replicates were then combined processed with Percolator (Käll et al., 2007). MSDaPl was used to visually inspect the results (Sharma et al., 2012) and confirm there was enough MS2 spectral evidence to differentiate between possible phosphorylated residues in a given peptide and give an accurate assignment. Because data dependent acquisition results in irreproducible sampling collection, additional mass spectrometry runs might reveal more phosphorylation events at each stage of the cell cycle.

## Supplementary Material

Supplementary information

## References

[BIO033647C1] Avena J. S., Burns S., Yu Z., Ebmeier C. C., Old W. M., Jaspersen S. L. and Winey M. (2014). Licensing of yeast centrosome duplication requires phosphoregulation of Sfi1. *PLoS Genet.* 10, e1004666 10.1371/journal.pgen.1004666 25340401 PMC4207612

[BIO033647C2] Basto R. and Pines J. (2007). The centrosome opens the way to mitosis. *Dev. Cell* 12, 475-477. 10.1016/j.devcel.2007.03.012 17419985

[BIO033647C3] Blondel M., Galan J. M., Chi Y., Lafourcade C., Longaretti C., Deshaies R. J. and Peter M. (2000). Nuclear-specific degradation of Far1 is controlled by the localization of the F-box protein Cdc4. *EMBO J.* 19, 6085-6097. 10.1093/emboj/19.22.6085 11080155 PMC305831

[BIO033647C4] Bouhlel I. B., Ohta M., Mayeux A., Bordes N., Dingli F., Boulanger J., Velve Casquillas G., Loew D., Tran P. T., Sato M. et al. (2015). Cell cycle control of spindle pole body duplication and splitting by Sfi1 and Cdc31 in fission yeast. *J. Cell Sci.* 128, 1481-1493. 10.1242/jcs.159657 25736294

[BIO033647C5] Bullitt E., Rout M. P., Kilmartin J. V. and Akey C. W. (1997). The yeast spindle pole body is assembled around a central crystal of Spc42p. *Cell* 89, 1077-1086. 10.1016/S0092-8674(00)80295-0 9215630

[BIO033647C6] Burke D. J., Dawson D. and Stearns T. (2000). *Methods in Yeast Genetics: A Cold Spring Harbor Laboratory Course Manual*. Cold Spring Harbor, USA: Cold Spring Harbor Laboratory Press.

[BIO033647C7] Burns S., Avena J. S., Unruh J. R., Yu Z., Smith S. E., Slaughter B. D., Winey M. and Jaspersen S. L. (2015). Structured illumination with particle averaging reveals novel roles for yeast centrosome components during duplication. *eLife* 4, e08586 10.7554/eLife.08586 PMC4564689 26371506

[BIO033647C8] Byers B. and Goetsch L. (1974). Duplication of spindle plaques and integration of the yeast cell cycle. *Cold Spring Harb. Symp. Quant. Biol.* 38, 123-131. 10.1101/SQB.1974.038.01.016 4598635

[BIO033647C9] Byers B. and Goetsch L. (1975). Behavior of spindles and spindle plaques in the cell cycle and conjugation of Saccharomyces cerevisiae. *J. Bacteriol.* 124, 511-523. 1100612 10.1128/jb.124.1.511-523.1975 PMC235921

[BIO033647C10] Casenghi M., Barr F. A. and Nigg E. A. (2005). Phosphorylation of Nlp by Plk1 negatively regulates its dynein-dynactin-dependent targeting to the centrosome. *J. Cell Sci.* 118, 5101-5108. 10.1242/jcs.02622 16254247

[BIO033647C11] Castillo A. R., Meehl J. B., Morgan G., Schutz-Geschwender A. and Winey M. (2002). The yeast protein kinase Mps1p is required for assembly of the integral spindle pole body component Spc42p. *J. Cell Biol.* 156, 453-465. 10.1083/jcb.200111025 11827982 PMC2173341

[BIO033647C12] Cha H., Hancock C., Dangi S., Maiguel D., Carrier F. and Shapiro P. (2004). Phosphorylation regulates nucleophosmin targeting to the centrosome during mitosis as detected by cross-reactive phosphorylation-specific MKK1/MKK2 antibodies. *Biochem. J.* 378, 857-865. 10.1042/bj20031173 14670079 PMC1224030

[BIO033647C13] Chambers M. C., Maclean B., Burke R., Amodei D., Ruderman D. L., Neumann S., Gatto L., Fischer B., Pratt B., Egertson J. et al. (2012). A cross-platform toolkit for mass spectrometry and proteomics. *Nat. Biotechnol.* 30, 918-920. 10.1038/nbt.2377 23051804 PMC3471674

[BIO033647C14] Deshaies R. J. and Ferrell J. E. Jr (2001). Multisite phosphorylation and the countdown to S phase. *Cell* 107, 819-822. 10.1016/S0092-8674(01)00620-1 11779457

[BIO033647C15] Duncker B. P., Pasero P., Braguglia D., Heun P., Weinreich M. and Gasser S. M. (1999). Cyclin B-Cdk1 kinase stimulates ORC- and Cdc6-independent steps of semiconservative plasmid replication in yeast nuclear extracts. *Mol. Cell. Biol.* 19, 1226-1241. 10.1128/MCB.19.2.1226 9891057 PMC116052

[BIO033647C16] Eng J. K., Jahan T. A. and Hoopmann M. R. (2013). Comet: an open-source MS/MS sequence database search tool. *Proteomics* 13, 22-24. 10.1002/pmic.201200439 23148064

[BIO033647C17] Flory M. R. and Davis T. N. (2003). The centrosomal proteins pericentrin and kendrin are encoded by alternatively spliced products of one gene. *Genomics* 82, 401-405. 10.1016/S0888-7543(03)00119-8 12906865

[BIO033647C18] Fong K. K., Graczyk B. and Davis T. N. (2016). Purification of fluorescently labeled saccharomyces cerevisiae spindle pole bodies. *Methods Mol. Biol.* 1413, 189-195. 10.1007/978-1-4939-3542-0_12 27193850 PMC4902105

[BIO033647C19] Friedman D. B., Sundberg H. A., Huang E. Y. and Davis T. N. (1996). The 110-kD spindle pole body component of Saccharomyces cerevisiae is a phosphoprotein that is modified in a cell cycle-dependent manner. *J. Cell Biol.* 132, 903-914. 10.1083/jcb.132.5.903 8603921 PMC2120732

[BIO033647C20] Friedman D. B., Kern J. W., Huneycutt B. J., Vinh D. B. N., Crawford D. K., Steiner E., Scheiltz D., Yates J. III, Resing K. A., Ahn N. G. et al. (2001). Yeast Mps1p phosphorylates the spindle pole component Spc110p in the N-terminal domain. *J. Biol. Chem.* 276, 17958-17967. 10.1074/jbc.M010461200 11278681 PMC4013285

[BIO033647C21] Gardner M. K., Bouck D. C., Paliulis L. V., Meehl J. B., O'Toole E. T., Haase J., Soubry A., Joglekar A. P., Winey M., Salmon E. D. et al. (2008). Chromosome congression by Kinesin-5 motor-mediated disassembly of longer kinetochore microtubules. *Cell* 135, 894-906. 10.1016/j.cell.2008.09.046 19041752 PMC2683758

[BIO033647C22] Gartner A., Jovanović A., Jeoung D.-I., Bourlat S., Cross F. R. and Ammerer G. (1998). Pheromone-dependent G1 cell cycle arrest requires Far1 phosphorylation, but may not involve inhibition of Cdc28-Cln2 kinase, in vivo. *Mol. Cell. Biol.* 18, 3681-3691. 10.1128/MCB.18.7.3681 9632750 PMC108950

[BIO033647C23] Greenberg C. H., Kollman J., Zelter A., Johnson R., Maccoss M. J., Davis T. N., Agard D. A. and Sali A. (2016). Structure of γ-tubulin small complex based on a cryo-EM map, chemical cross-links, and a remotely related structure. *J. Struct. Biol.* 194, 303-310. 10.1016/j.jsb.2016.03.006 26968363 PMC4866596

[BIO033647C24] Huisman S. M., Smeets M. F. M. A. and Segal M. (2007). Phosphorylation of Spc110p by Cdc28p-Clb5p kinase contributes to correct spindle morphogenesis in S. cerevisiae. *J. Cell Sci.* 120, 435-446. 10.1242/jcs.03342 17213332

[BIO033647C25] Jaspersen S. L. and Winey M. (2004). The budding yeast spindle pole body: structure, duplication, and function. *Annu. Rev. Cell Dev. Biol.* 20, 1-28. 10.1146/annurev.cellbio.20.022003.114106 15473833

[BIO033647C26] Jaspersen S. L., Huneycutt B. J., Giddings T. H., Resing K. A., Ahn N. G. and Winey M. (2004). Cdc28/Cdk1 regulates spindle pole body duplication through phosphorylation of Spc42 and Mps1. *Dev. Cell* 7, 263-274. 10.1016/j.devcel.2004.07.006 15296722

[BIO033647C27] Jeoung D.-I., Oehlen L. J. W. M. and Cross F. R. (1998). Cln3-associated kinase activity in Saccharomyces cerevisiae is regulated by the mating factor pathway. *Mol. Cell. Biol.* 18, 433-441. 10.1128/MCB.18.1.433 9418890 PMC121512

[BIO033647C28] Jiang N., Wang X., Jhanwar-Uniyal M., Darzynkiewicz Z. and Dai W. (2006). Polo box domain of Plk3 functions as a centrosome localization signal, overexpression of which causes mitotic arrest, cytokinesis defects, and apoptosis. *J. Biol. Chem.* 281, 10577-10582. 10.1074/jbc.M513156200 16478733

[BIO033647C29] Käll L., Canterbury J. D., Weston J., Noble W. S. and MacCoss M. J. (2007). Semi-supervised learning for peptide identification from shotgun proteomics datasets. *Nat. Methods* 4, 923-925. 10.1038/nmeth1113 17952086

[BIO033647C30] Keck J. M., Jones M. H., Wong C. C. L., Binkley J., Chen D., Jaspersen S. L., Holinger E. P., Xu T., Niepel M., Rout M. P. et al. (2011). A cell cycle phosphoproteome of the yeast centrosome. *Science* 332, 1557-1561. 10.1126/science.1205193 21700874 PMC3825980

[BIO033647C31] Lange B. M. H. (2002). Integration of the centrosome in cell cycle control, stress response and signal transduction pathways. *Curr. Opin. Cell Biol.* 14, 35-43. 10.1016/S0955-0674(01)00291-5 11792542

[BIO033647C32] Lin T., Gombos L., Neuner A., Sebastian D., Olsen J. V., Hrle A., Benda C. and Schiebel E. (2011). Phosphorylation of the yeast γ-tubulin Tub4 regulates microtubule function. *PLoS ONE* 6, e19700 10.1371/journal.pone.0019700 21573187 PMC3088709

[BIO033647C33] Lin T., Neuner A., Schlosser Y. T., Scharf A. N., Weber L. and Schiebel E. (2014). Cell-cycle dependent phosphorylation of yeast pericentrin regulates γ-TuSC-mediated microtubule nucleation. *eLife* 3, e02208 10.7554/eLife.02208 24842996 PMC4034690

[BIO033647C34] Muller E. G. D., Snydsman B. E., Novik I., Hailey D. W., Gestaut D. R., Niemann C. A., O'Toole E. T., Giddings T. H., Sundin B. A. and Davis T. N. (2005). The organization of the core proteins of the yeast spindle pole body. *Mol. Biol. Cell* 16, 3341-3352. 10.1091/mbc.e05-03-0214 15872084 PMC1165416

[BIO033647C35] Niepel M., Strambio-de-Castillia C., Fasolo J., Chait B. T. and Rout M. P. (2005). The nuclear pore complex-associated protein, Mlp2p, binds to the yeast spindle pole body and promotes its efficient assembly. *J. Cell Biol.* 170, 225-235. 10.1083/jcb.200504140 16027220 PMC2171418

[BIO033647C36] Oakley B. R., Oakley C. E., Yoon Y. and Jung M. K. (1990). γ-tubulin is a component of the spindle pole body that is essential for microtubule function in Aspergillus nidulans. *Cell* 61, 1289-1301. 10.1016/0092-8674(90)90693-9 2194669

[BIO033647C37] O'Toole E. T., Winey M. and McIntosh J. R. (1999). High-voltage electron tomography of spindle pole bodies and early mitotic spindles in the yeast Saccharomyces cerevisiae. *Mol. Biol. Cell* 10, 2017-2031. 10.1091/mbc.10.6.2017 10359612 PMC25406

[BIO033647C38] Peng Y., Moritz M., Han X., Giddings T. H., Lyon A., Kollman J., Winey M., Yates J., Agard D. A., Drubin D. G. et al. (2015). Interaction of CK1δ with γTuSC ensures proper microtubule assembly and spindle positioning. *Mol. Biol. Cell* 26, 2505-2518. 10.1091/mbc.e14-12-1627 25971801 PMC4571304

[BIO033647C39] Pereira G., Knop M. and Schiebel E. (1998). Spc98p directs the yeast γ-tubulin complex into the nucleus and is subject to cell cycle-dependent phosphorylation on the nuclear side of the spindle pole body. *Mol. Biol. Cell* 9, 775-793. 10.1091/mbc.9.4.775 9529377 PMC25305

[BIO033647C40] Peter M. and Herskowitz I. (1994). Direct inhibition of the yeast cyclin-dependent kinase Cdc28-Cln by Far1. *Science* 265, 1228-1231. 10.1126/science.8066461 8066461

[BIO033647C41] Rieder C. L., Faruki S. and Khodjakov A. (2001). The centrosome in vertebrates: more than a microtubule-organizing center. *Trends Cell Biol.* 11, 413-419. 10.1016/S0962-8924(01)02085-2 11567874

[BIO033647C42] Sharma V., Eng J. K., Maccoss M. J. and Riffle M. (2012). A mass spectrometry proteomics data management platform. *Mol. Cell. Proteomics* 11, 824-831. 10.1074/mcp.O111.015149 22611296 PMC3434774

[BIO033647C43] Shimogawa M. M., Widlund P. O., Riffle M., Ess M. and Davis T. N. (2009). Bir1 is required for the tension checkpoint. *Mol. Biol. Cell* 20, 915-923. 10.1091/mbc.e08-07-0723 19056681 PMC2633401

[BIO033647C44] Sikorski R. S. and Hieter P. (1989). A system of shuttle vectors and yeast host strains designed for efficient manipulation of DNA in Saccharomyces cerevisiae. *Genetics* 122, 19-27. 2659436 10.1093/genetics/122.1.19 PMC1203683

[BIO033647C45] Stearns T., Evans L. and Kirschner M. (1991). γ-tubulin is a highly conserved component of the centrosome. *Cell* 65, 825-836. 10.1016/0092-8674(91)90390-K 1840506

[BIO033647C46] Stirling D. A. and Stark M. J. R. (1996). The phosphorylation state of the 110 kDa component of the yeast spindle pole body shows cell cycle dependent regulation. *Biochem. Biophys. Res. Commun.* 222, 236-242. 10.1006/bbrc.1996.0728 8670189

[BIO033647C47] Sundberg H. A., Goetsch L., Byers B. and Davis T. N. (1996). Role of calmodulin and Spc110p interaction in the proper assembly of spindle pole body components. *J. Cell Biol.* 133, 111-124. 10.1083/jcb.133.1.111 8601600 PMC2120774

[BIO033647C48] Tien J. F., Fong K. K., Umbreit N. T., Payen C., Zelter A., Asbury C. L., Dunham M. J. and Davis T. N. (2013). Coupling unbiased mutagenesis to high-throughput DNA sequencing uncovers functional domains in the Ndc80 kinetochore protein of Saccharomyces cerevisiae. *Genetics* 195, 159-170. 10.1534/genetics.113.152728 23833183 PMC3761298

[BIO033647C49] Viswanath S., Bonomi M., Kim S. J., Klenchin V. A., Taylor K. C., Yabut K. C., Umbreit N. T., Van Epps H. A., Meehl J., Jones M. H. et al. (2017). The molecular architecture of the yeast spindle pole body core determined by Bayesian integrative modeling. *Mol. Biol. Cell* 28, 3298-3314. 10.1091/mbc.e17-06-0397 28814505 PMC5687031

[BIO033647C50] Vogel J., Drapkin B., Oomen J., Beach D., Bloom K. and Snyder M. (2001). Phosphorylation of γ-tubulin regulates microtubule organization in budding yeast. *Dev. Cell* 1, 621-631. 10.1016/S1534-5807(01)00073-9 11709183

[BIO033647C51] Widlund P. O. and Davis T. N. (2005). A high-efficiency method to replace essential genes with mutant alleles in yeast. *Yeast* 22, 769-774. 10.1002/yea.1244 16088871 PMC1698466

[BIO033647C52] Winey M. and Bloom K. (2012). Mitotic spindle form and function. *Genetics* 190, 1197-1224. 10.1534/genetics.111.128710 22491889 PMC3316638

[BIO033647C53] Winey M., Goetsch L., Baum P. and Byers B. (1991). MPS1 and MPS2: novel yeast genes defining distinct steps of spindle pole body duplication. *J. Cell Biol.* 114, 745-754. 10.1083/jcb.114.4.745 1869587 PMC2289888

[BIO033647C54] Woodruff J. B., Wueseke O. and Hyman A. A. (2014). Pericentriolar material structure and dynamics. *Philos. Trans. R. Soc. Lond. B Biol. Sci.* 369, 20130459 10.1098/rstb.2013.0459 25047613 PMC4113103

[BIO033647C55] Woodruff J. B., Wueseke O., Viscardi V., Mahamid J., Ochoa S. D., Bunkenborg J., Widlund P. O., Pozniakovsky A., Zanin E., Bahmanyar S. et al. (2015). Centrosomes. Regulated assembly of a supramolecular centrosome scaffold in vitro. *Science* 348, 808-812. 10.1126/science.aaa3923 25977552 PMC5039038

