# Supplementary material for: Novel phosphorylation states of the yeast spindle pole body
Source: Biol Open. 2018 Jun 14;7(10):bio033647. doi: 10.1242/bio.033647 (PMC6215409; doi:10.1242/bio.033647)
Supplement: Supplementary information [file biolopen-7-033647-s1.pdf]

NOVEL PHOSPHORYLATION STATES OF THE YEAST SPINDLE POLE BODY: SUPPLEMENTAL MATERIALS

Kimberly K. Fong[1], Alex Zelter[1], Beth Graczyk[1], Jill Hoyt[1], Michael Riffle[1], Richard Johnson[2], Michael J. MacCoss[2], Trisha N. Davis[1]

[1]Department of Biochemistry, University of Washington, Seattle, WA 98195, USA

[2]Department of Genome Sciences, University of Washington, Seattle, WA 98195, USA

Corresponding author: tdavis@uw.edu

Supplemental Table 1.

The percent of PSMs and spectra for the mass spectrometry data sets with a q-value <=0.01, as calculated by Percolator statistical package.

	**qvalue <=0.01 (%)**	
	**PSM**	**spectra**
**Asynchronous SPBs**	57.6	47.0
	60.5	27.7
	50.5	36.6
**CDC20-AID SPBs**	65.9	22.8
	39.0	5.9
	46.0	35.4
**cdc4-1 SPBs**	52.8	37.8
	62.1	47.6

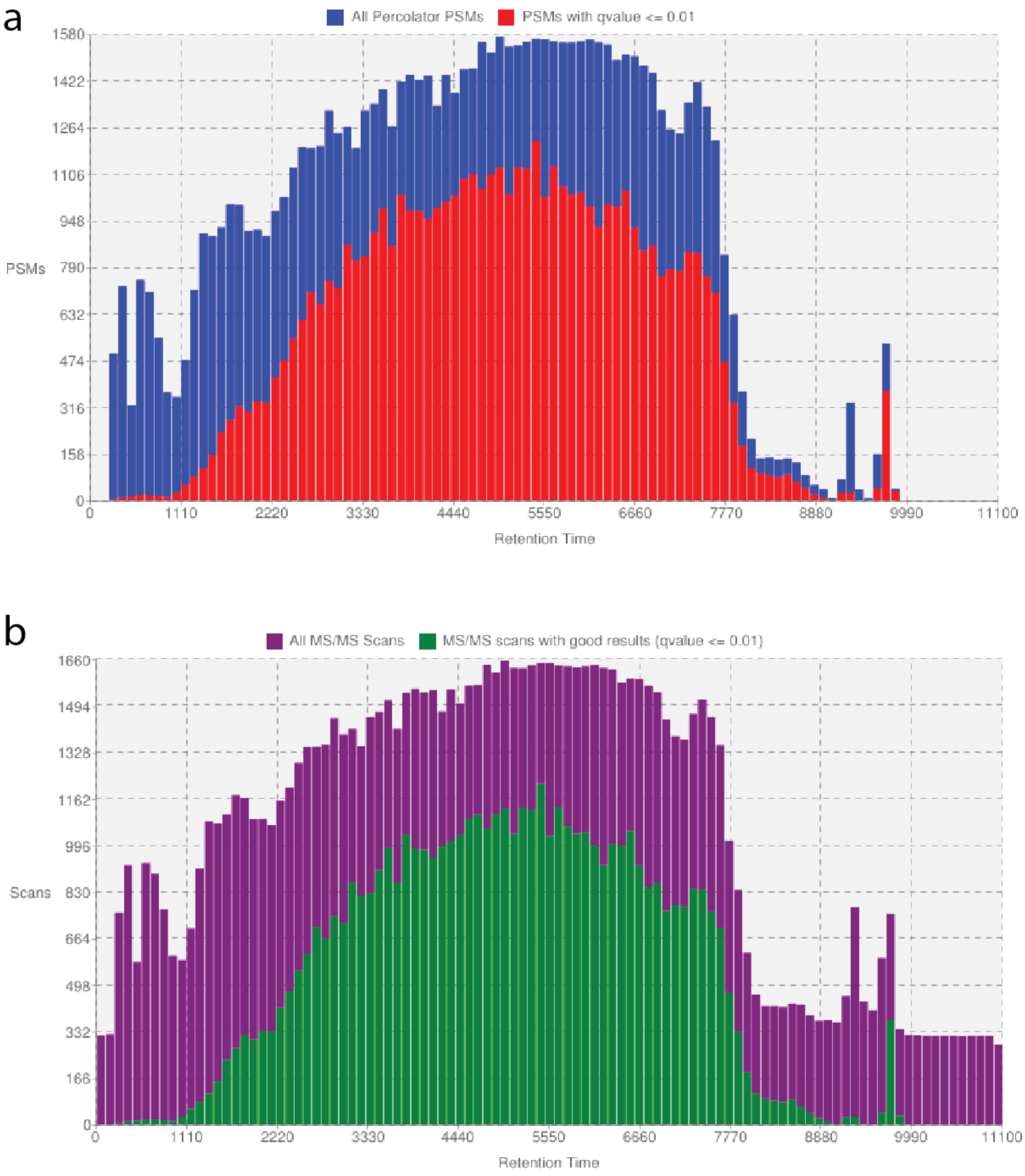

Supplemental Figure 1. A graphical representation of the quality of PSMs and spectra. a. The total number of PSMs are graphed in blue, while the high quality PSMs (q-value <=0.01) are graphed in red. b. The total number of spectra are graphed in purple, while the high quality spectra (q-value <=0.01) are graphed in green.

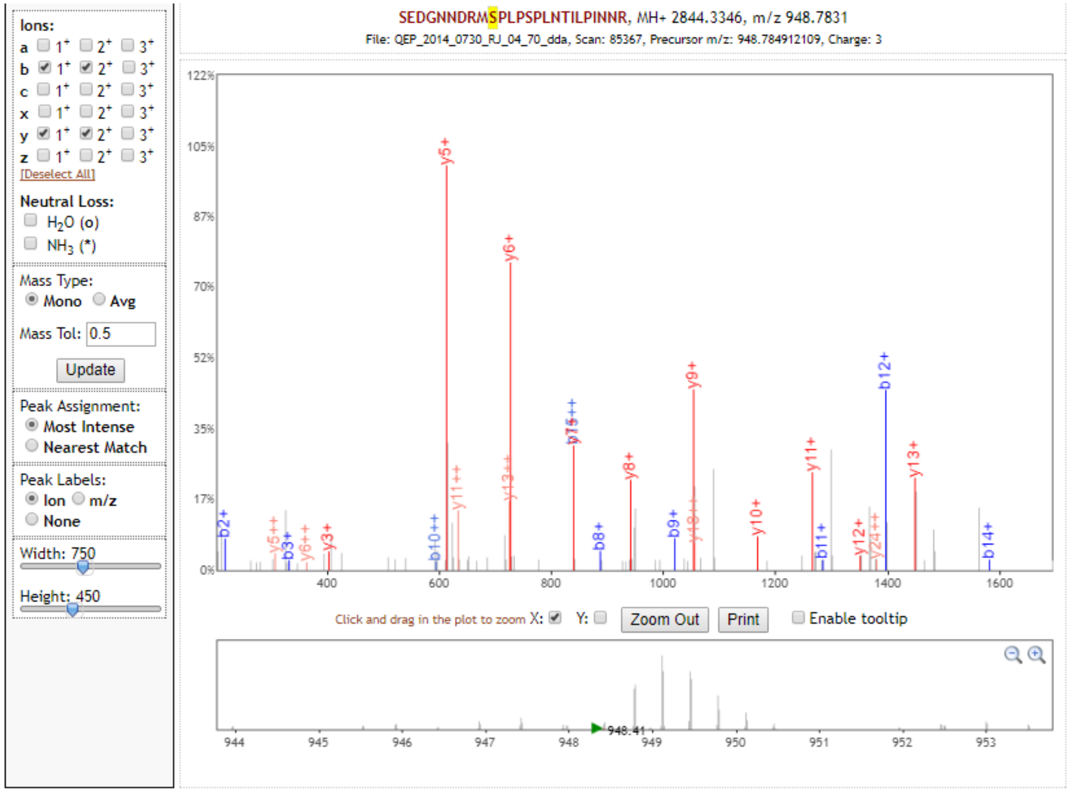

b+	b2+	#	Seq	#	y+	y2+
88.0393	44.5233	1	S	25		
217.0819	109.0446	2	E	24	2757.3026	1379.1549
332.1088	166.5581	3	D	23	2628.2600	1314.6336
389.1303	195.0688	4	G	22	2513.2330	1257.1202
503.1732	252.0903	5	N	21	2456.2116	1228.6094
617.2162	309.1117	6	N	20	2342.1686	1171.5880
732.2431	366.6252	7	D	19	2228.1257	1114.5665
888.3442	444.6757	8	R	18	2113.0988	1057.0530
1019.3847	510.1960	9	M	17	1956.9977	979.0025
1186.3831	593.6952	10	S	16	1825.9572	913.4822
1283.4358	642.2215	11	P	15	1658.9588	829.9830
1396.5199	698.7636	12	L	14	1561.9061	781.4567
1493.5726	747.2900	13	P	13	1448.8220	724.9146
1580.6047	790.8060	14	S	12	1351.7692	676.3883
1677.6574	839.3324	15	P	11	1264.7372	632.8722
1790.7415	895.8744	16	L	10	1167.6844	584.3459
1904.7844	952.8959	17	N	9	1054.6004	527.8038
2005.8321	1003.4197	18	T	8	940.5574	470.7824
2118.9162	1059.9617	19	I	7	839.5098	420.2585
2232.0002	1116.5038	20	L	6	726.4257	363.7165
2329.0530	1165.0301	21	P	5	613.3416	307.1745
2442.1371	1221.5722	22	I	4	516.2889	258.6481
2556.1800	1278.5936	23	N	3	403.2048	202.1060
2670.2229	1335.6151	24	N	2	289.1619	145.0846
		25	R	1	175.1190	88.0631

[Click] to move table

Static Modifications:
C: 57.021464

Variable Modifications:
S: 79.966331

Obs. Mass	Calc. Mass	Charge	RT	XCr	XCorr	dCN	e-value	Peptide
2844.34	2844.33	3	7108.43	1	2.97	0.00000	0.0	(J) (JS) R.SEDGNNDRMS[+80]PLPSPLNTILPINNR.L

Supplemental Figure 2. Example of MS2 spectrum from Spc42. The main box in the center represents the MS2 spectrum with peak assignments. The table to the right displays the calculated values of b and y ions, where the color shaded boxes represent fragment ion peaks that were identified in the MS2 spectrum. The graphical representation was generated using Lorikeet software http://uwpr.github.io/Lorikeet/.

Biology Open • Supplementary information

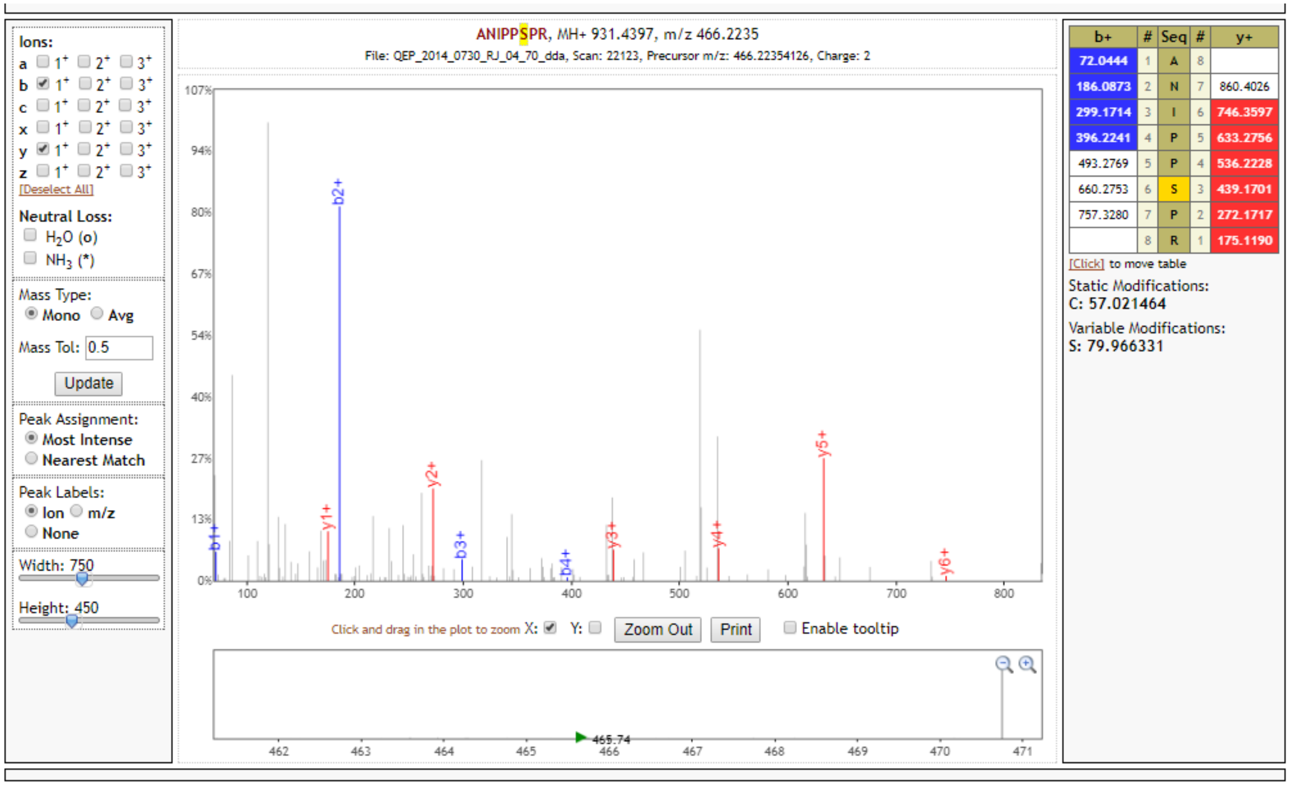

Obs. Mass	Calc. Mass	Charge	RT	XCr	XCorr	dCN	e-value	Peptide
931.44	931.44	2	2628.01	1	1.27	0.00000	0.0127	(J) (JS) K.ANIPPS[+80]PR.S

Supplemental Figure 3. Example of MS2 spectrum from Spc110. The main box in the center represents the MS2 spectrum with peak assignments. The table to the right displays the calculated values of b and y ions, where the color shaded boxes represent fragment ion peaks that were identified in the MS2 spectrum. The graphical representation was generated using Lorikeet software http://uwpr.github.io/Lorikeet/.

Bbp1 Asynchronous

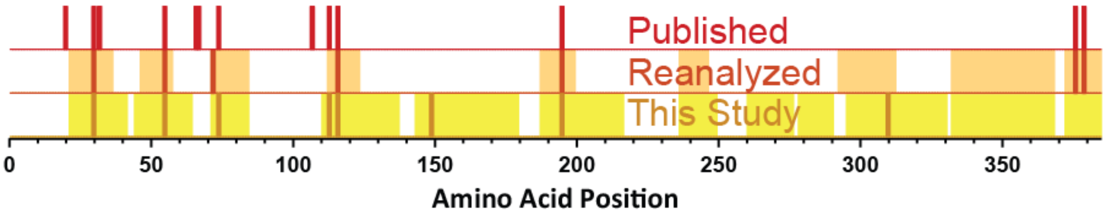

Bbp1 Mitotic

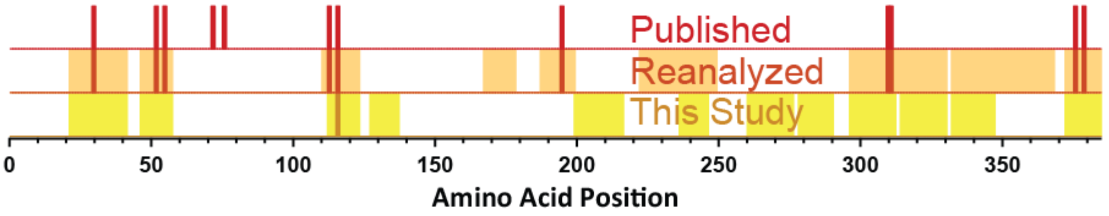

Bbp1 G1

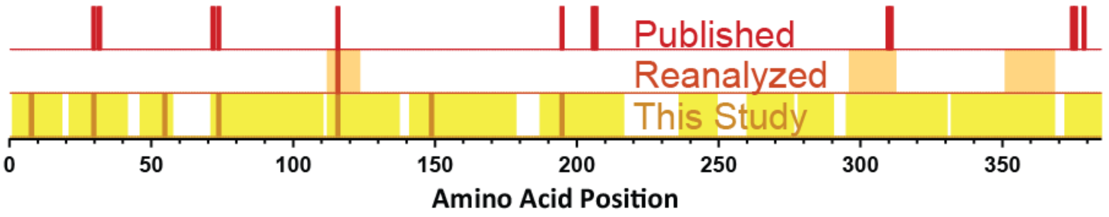

Cdc31 Asynchronous

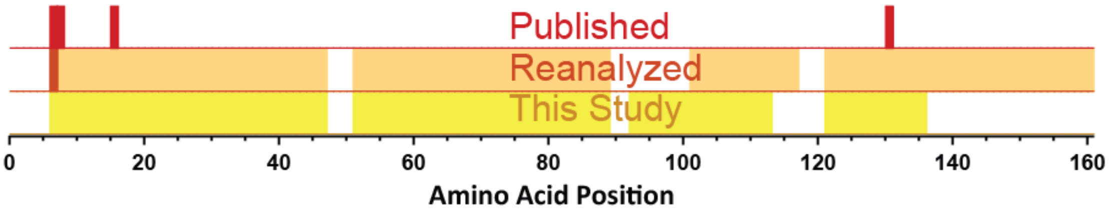

Cdc31 Mitotic

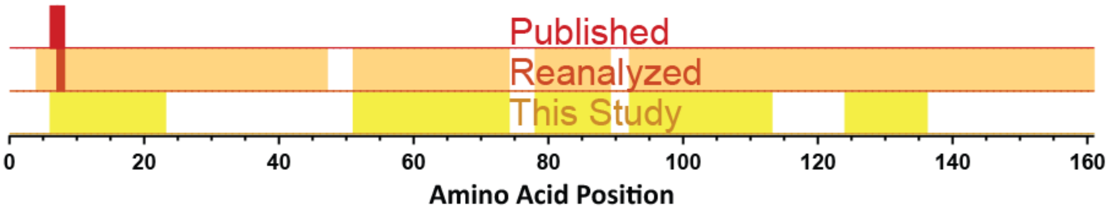

Cdc31 G1

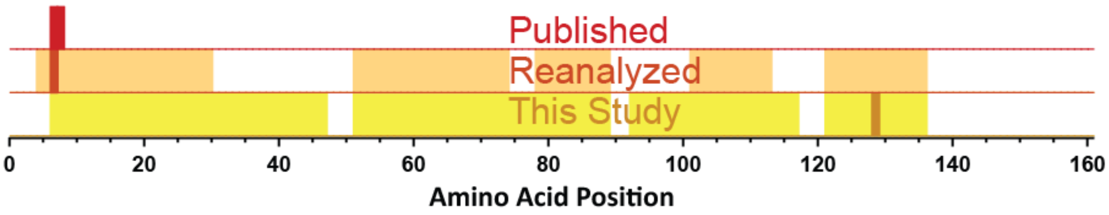

Biology Open • Supplementary information

Supplemental Figure 4. Comparison of Bbp1 and Cdc31 asynchronous, mitotic, and G1 phosphorylation sites. Published phosphorylation sites from Keck *et al*, 2011 are indicated as red bars. Peptide coverage of the reanalyzed Keck *et al*, 2011 data set and this data set are shown in the light orange and yellow shadings, respectively. Identified phosphorylation sites for the reanalyzed Keck *et al*, 2011 data set are indicated by the dark orange, and identified phosphorylation sites from this study are indicated by dark yellow bars. Note that the G1 data sets from Keck *et al*, 2011 were collected from SPBs isolated from cells arrested in alpha factor, while the G1 data set from this study was collected from SPBs isolated from cells arrested using a *cdc4-1* temperature sensitive mutation.

Cmd1 Asynchronous

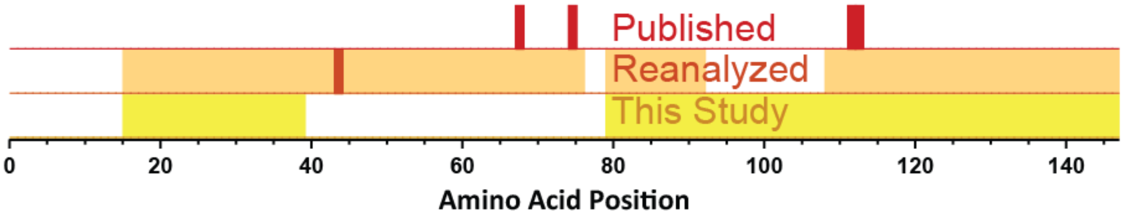

Cmd1 Mitotic

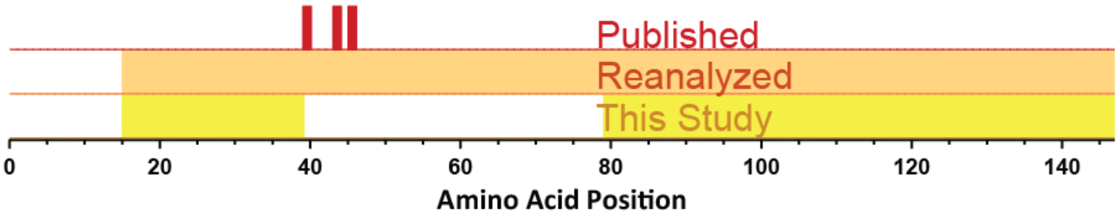

Cmd1 G1

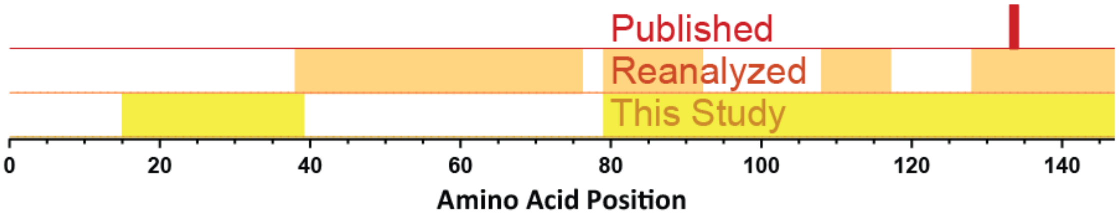

Cnm67 Asynchronous

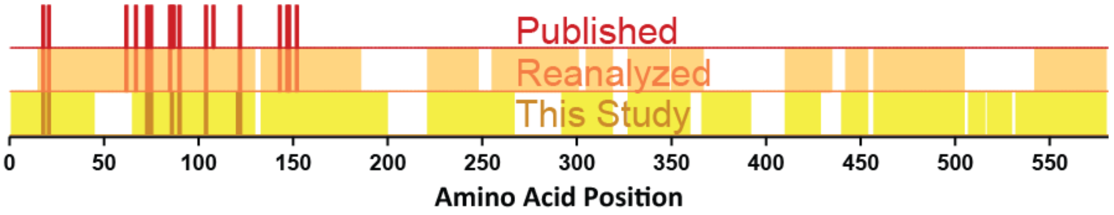

Cnm67 Mitotic

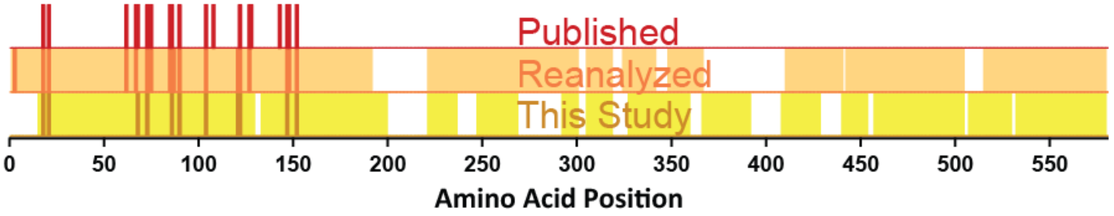

Cnm67 G1

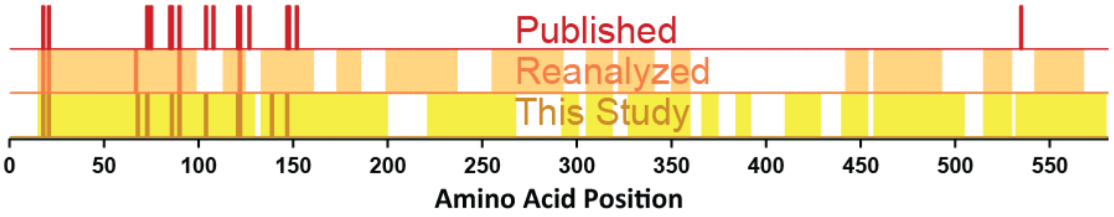

Biology Open • Supplementary information

Supplemental Figure 5. Comparison of Cmd1 and Cnm67 asynchronous, mitotic, and G1 phosphorylation sites. Published phosphorylation sites from Keck *et al*, 2011 are indicated as red bars. Peptide coverage of the reanalyzed Keck *et al*, 2011 data set and this data set are shown in the light orange and yellow shadings, respectively. Identified phosphorylation sites for the reanalyzed Keck *et al*, 2011 data set are indicated by the dark orange, and identified phosphorylation sites from this study are indicated by dark yellow bars. Note that the G1 data sets from Keck *et al*, 2011 were collected from SPBs isolated from cells arrested in alpha factor, while the G1 data set from this study was collected from SPBs isolated from cells arrested using a *cdc4-1* temperature sensitive mutation.

Kar1 Asynchronous

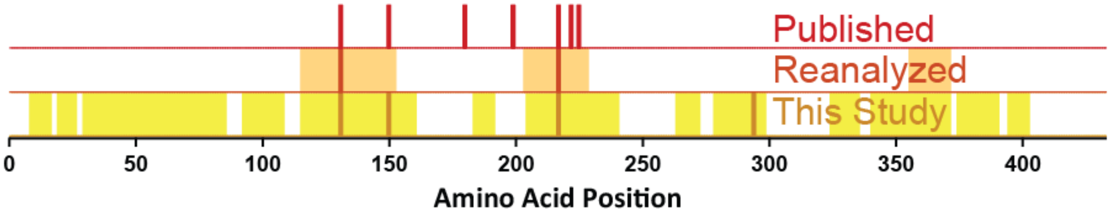

Kar1 Mitotic

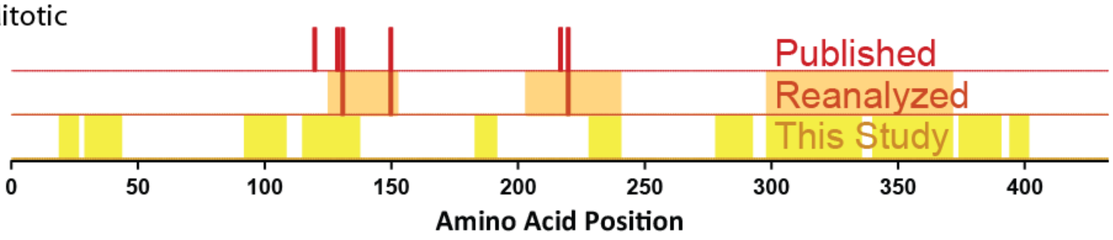

Kar1 G1

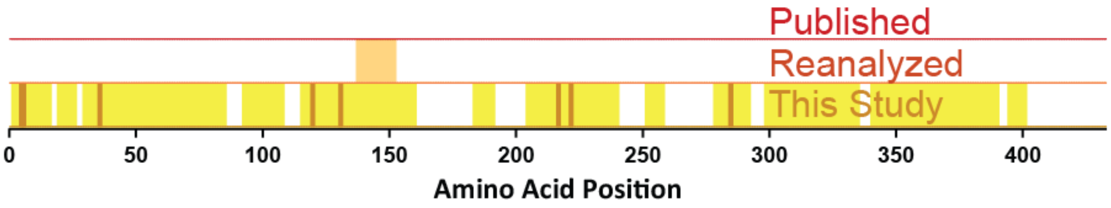

Mps2 Asynchronous

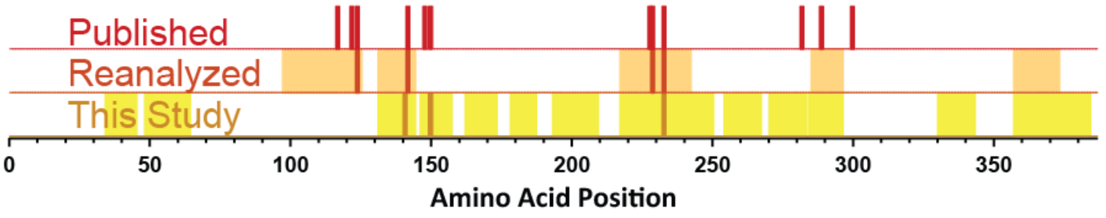

Mps2 Mitotic

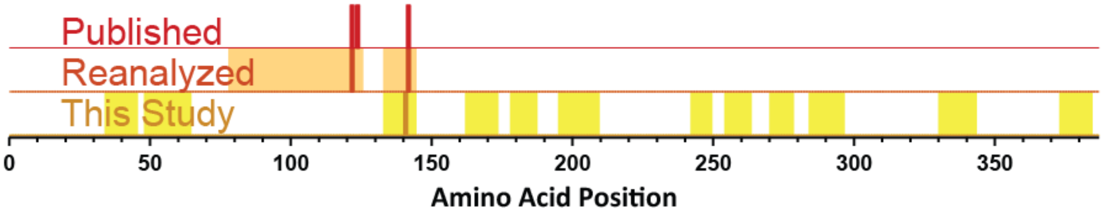

Mps2 G1

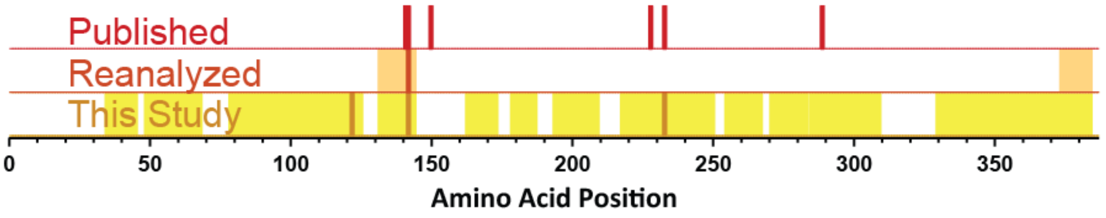

Biology Open • Supplementary information

Supplemental Figure 6. Comparison of Kar1 and Mps2 asynchronous, mitotic, and G1 phosphorylation sites. Published phosphorylation sites from Keck *et al*, 2011 are indicated as red bars. Peptide coverage of the reanalyzed Keck *et al*, 2011 data set and this data set are shown in the light orange and yellow shadings, respectively. Identified phosphorylation sites for the reanalyzed Keck *et al*, 2011 data set are indicated by the dark orange, and identified phosphorylation sites from this study are indicated by dark yellow bars. Note that the G1 data sets from Keck *et al*, 2011 were collected from SPBs isolated from cells arrested in alpha factor, while the G1 data set from this study was collected from SPBs isolated from cells arrested using a *cdc4-1* temperature sensitive mutation.

Biology Open • Supplementary information

Mps3 Asynchronous

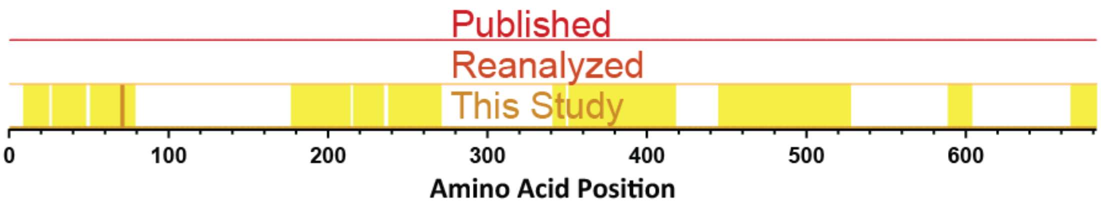

Mps3 Mitotic

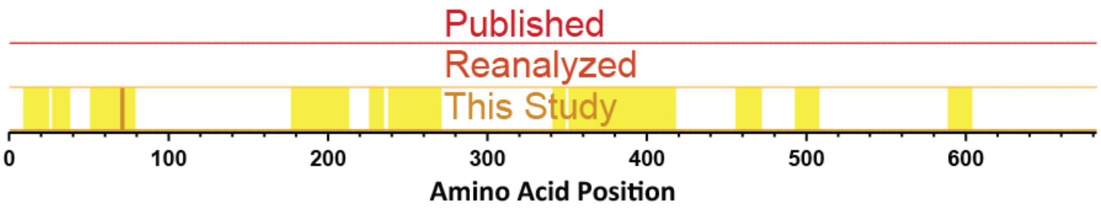

Mps3 G1

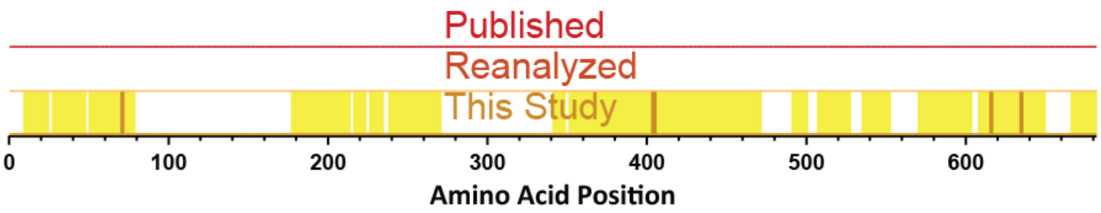

Nbp1 Asynchronous

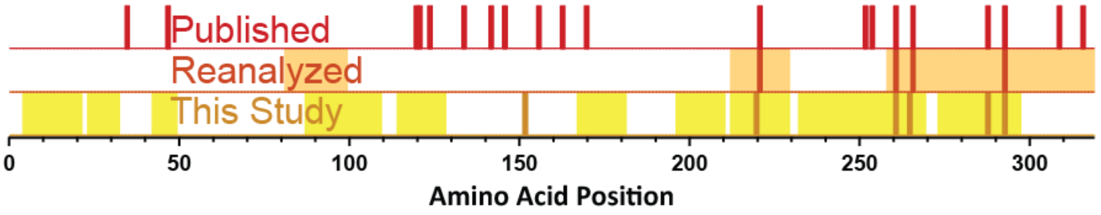

Nbp1 Mitotic

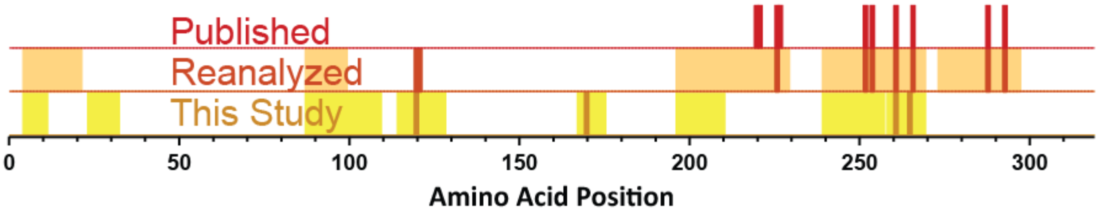

Nbp1 G1

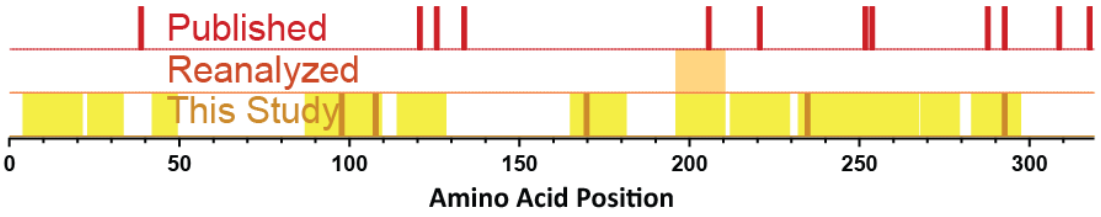

Supplemental Figure 7. Comparison of Mps3 and Nbp1 asynchronous, mitotic, and G1 phosphorylation sites. Published phosphorylation sites from Keck *et al*, 2011 are indicated as red bars. Peptide coverage of the reanalyzed Keck *et al*, 2011 data set and this data set are shown in the light orange and yellow shadings, respectively. Identified phosphorylation sites for the reanalyzed Keck *et al*, 2011 data set are indicated by the dark orange, and identified phosphorylation sites from this study are indicated by dark yellow bars. Note that the G1 data sets from Keck *et al*, 2011 were collected from SPBs isolated from cells arrested in alpha factor, while the G1 data set from this study was collected from SPBs isolated from cells arrested using a *cdc4-1* temperature sensitive mutation.

Biology Open • Supplementary information

Ndc1 Asynchronous

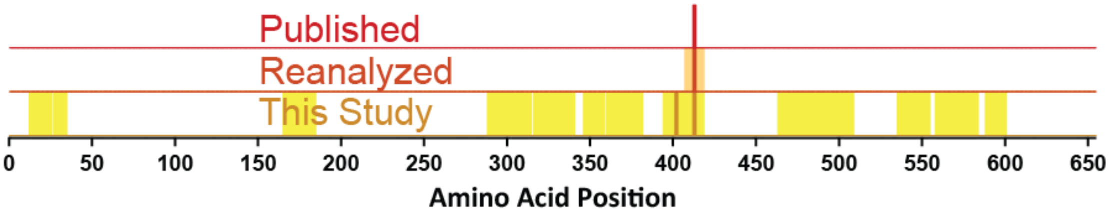

Ndc1 Mitotic

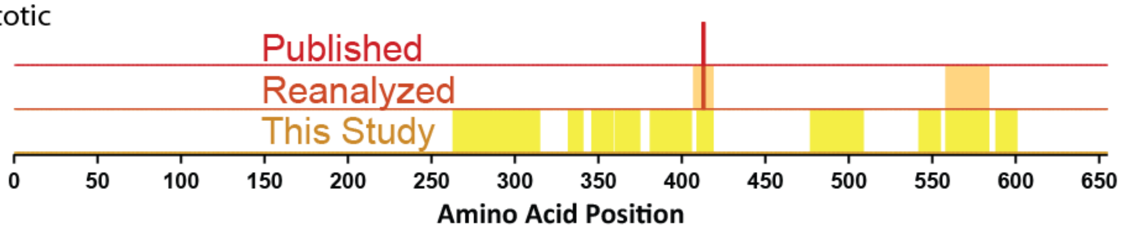

Ndc1 G1

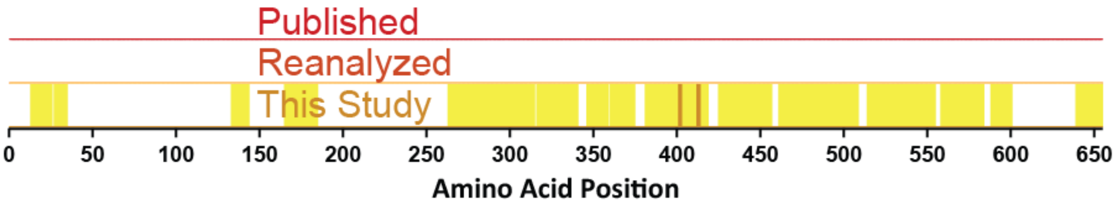

Nud1 Asynchronous

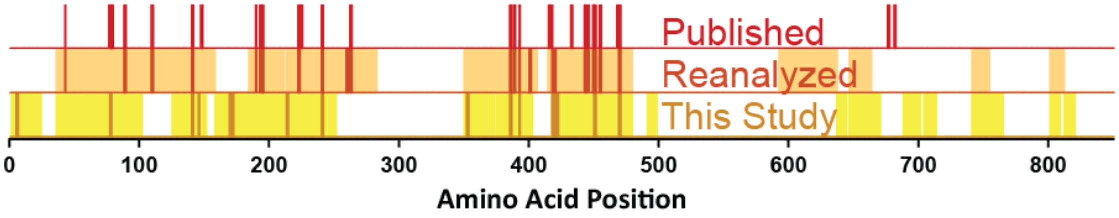

Nud1 Mitotic

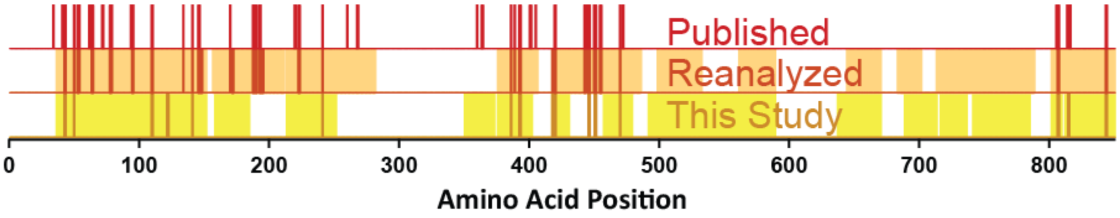

Nud1 G1

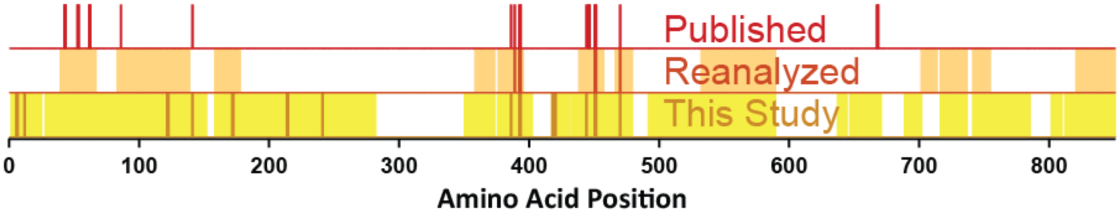

Biology Open • Supplementary information

Supplemental Figure 8. Comparison of Ndc1 and Nud1 asynchronous, mitotic, and G1 phosphorylation sites. Published phosphorylation sites from Keck *et al*, 2011 are indicated as red bars. Peptide coverage of the reanalyzed Keck *et al*, 2011 data set and this data set are shown in the light orange and yellow shadings, respectively. Identified phosphorylation sites for the reanalyzed Keck *et al*, 2011 data set are indicated by the dark orange, and identified phosphorylation sites from this study are indicated by dark yellow bars. Note that the G1 data sets from Keck *et al*, 2011 were collected from SPBs isolated from cells arrested in alpha factor, while the G1 data set from this study was collected from SPBs isolated from cells arrested using a *cdc4-1* temperature sensitive mutation.

Sfi1 Asynchronous

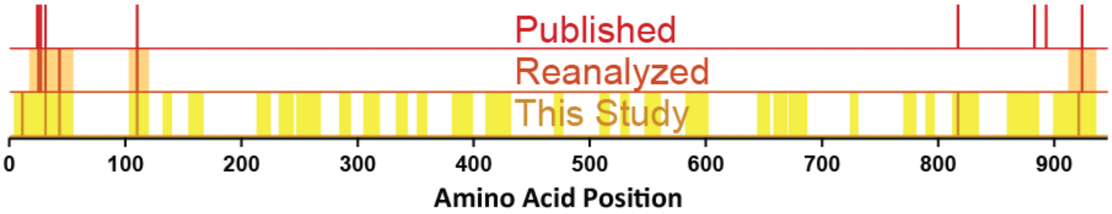

Sfi1 Mitotic

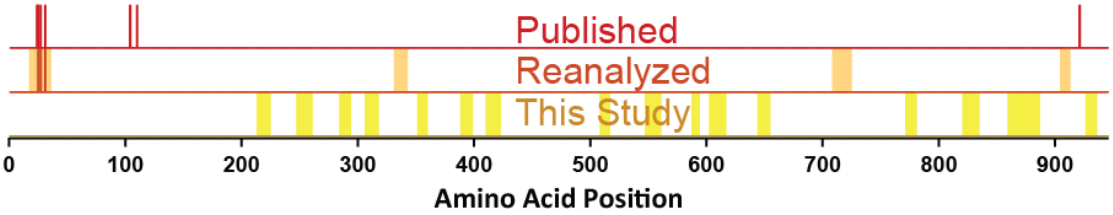

Sfi1 G1

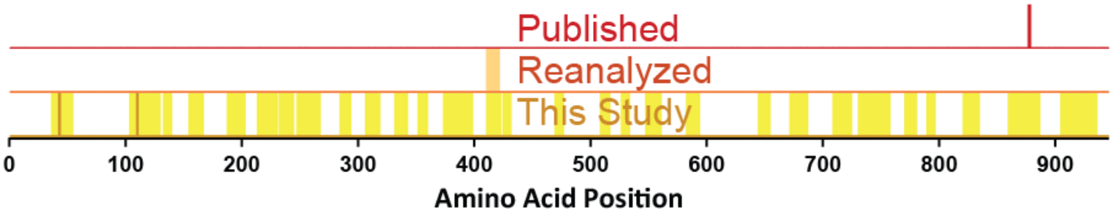

Spc29 Asynchronous

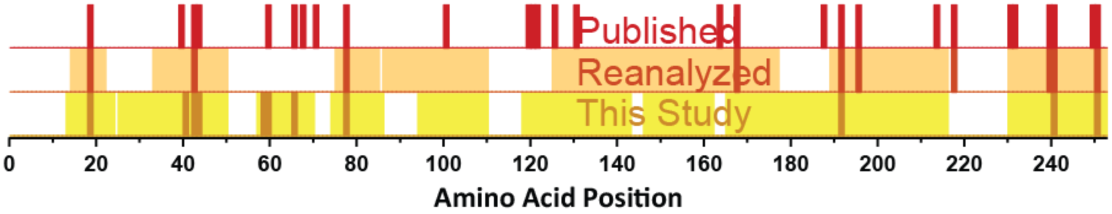

Spc29 Mitotic

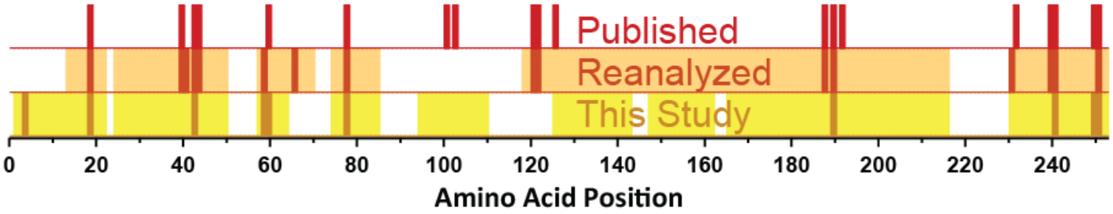

Spc29 G1

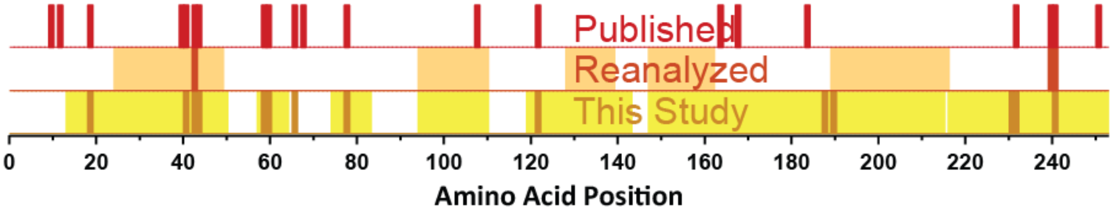

Biology Open • Supplementary information

Supplemental Figure 9. Comparison of Sfi1 and Spc29 asynchronous, mitotic, and G1 phosphorylation sites. Published phosphorylation sites from Keck *et al*, 2011 are indicated as red bars. Peptide coverage of the reanalyzed Keck *et al*, 2011 data set and this data set are shown in the light orange and yellow shadings, respectively. Identified phosphorylation sites for the reanalyzed Keck *et al*, 2011 data set are indicated by the dark orange, and identified phosphorylation sites from this study are indicated by dark yellow bars. Note that the G1 data sets from Keck *et al*, 2011 were collected from SPBs isolated from cells arrested in alpha factor, while the G1 data set from this study was collected from SPBs isolated from cells arrested using a *cdc4-1* temperature sensitive mutation.

Spc42 Asynchronous

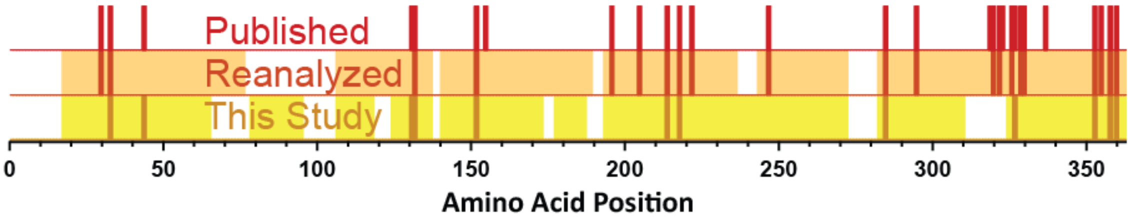

Spc42 Mitotic

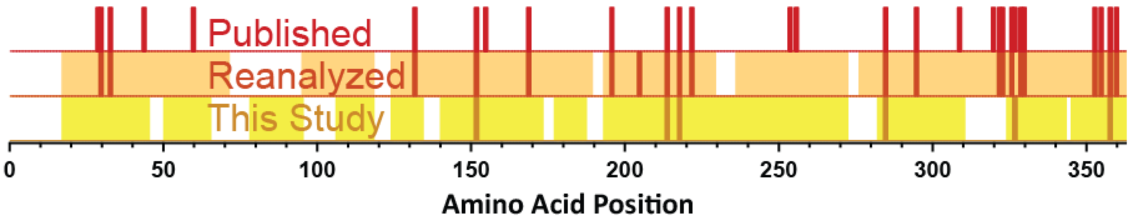

Spc42 G1

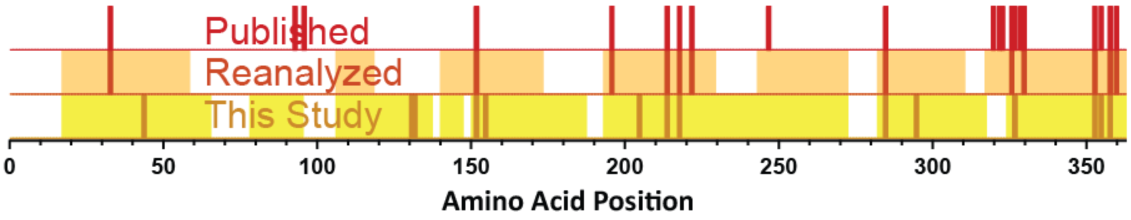

Spc72 Asynchronous

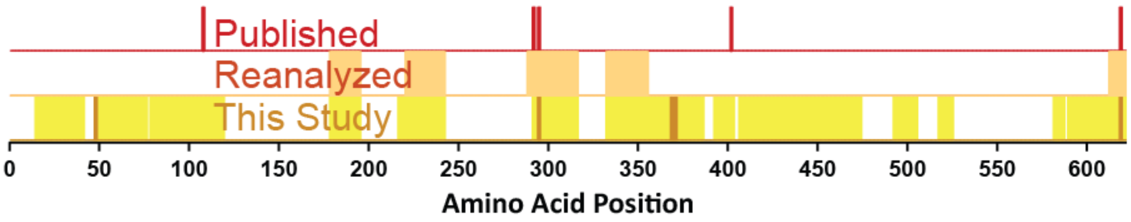

Spc72 Mitotic

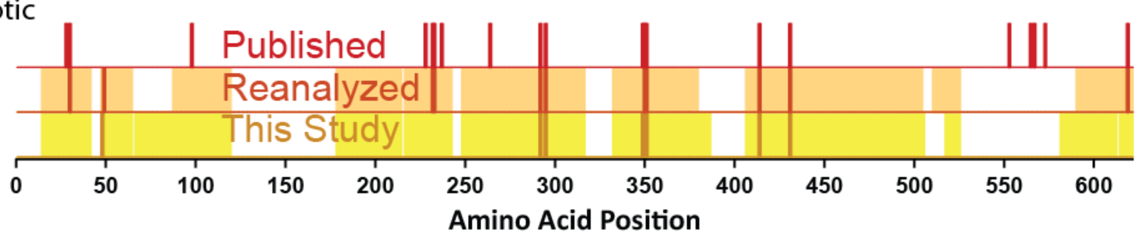

Spc72 G1

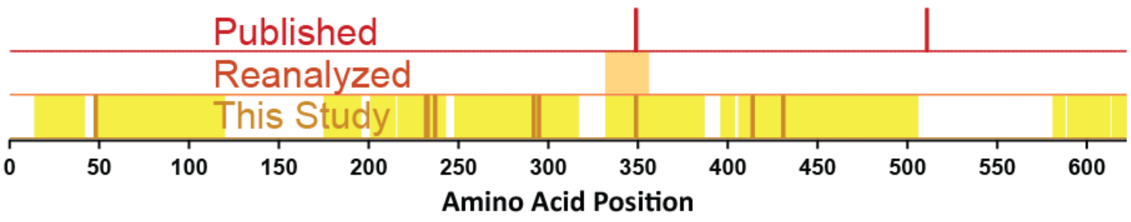

Biology Open • Supplementary information

Supplemental Figure 10. Comparison of Spc42 and Spc72 asynchronous, mitotic, and G1 phosphorylation sites. Published phosphorylation sites from Keck *et al*, 2011 are indicated as red bars. Peptide coverage of the reanalyzed Keck *et al*, 2011 data set and this data set are shown in the light orange and yellow shadings, respectively. Identified phosphorylation sites for the reanalyzed Keck *et al*, 2011 data set are indicated by the dark orange, and identified phosphorylation sites from this study are indicated by dark yellow bars. Note that the G1 data sets from Keck *et al*, 2011 were collected from SPBs isolated from cells arrested in alpha factor, while the G1 data set from this study was collected from SPBs isolated from cells arrested using a *cdc4-1* temperature sensitive mutation.

Spc97 Asynchronous

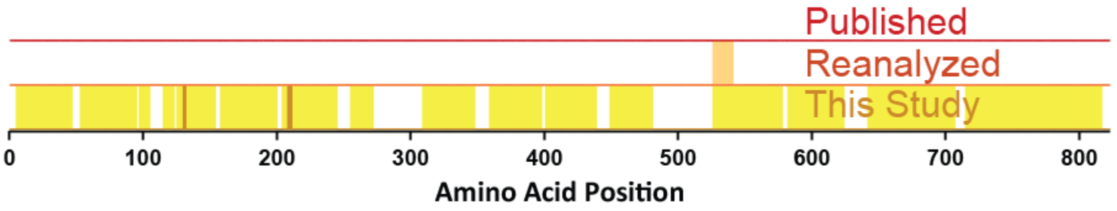

Spc97 Mitotic

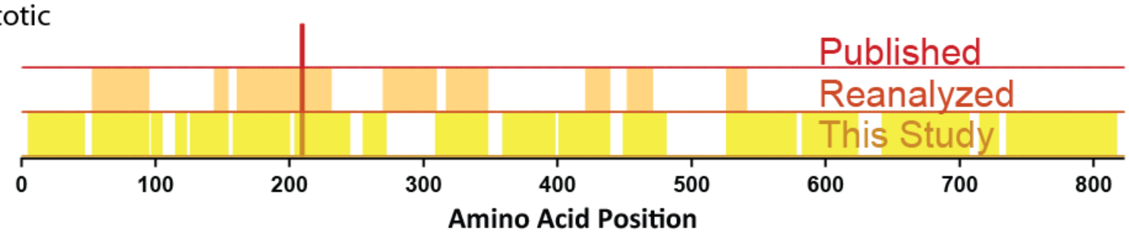

Spc97 G1

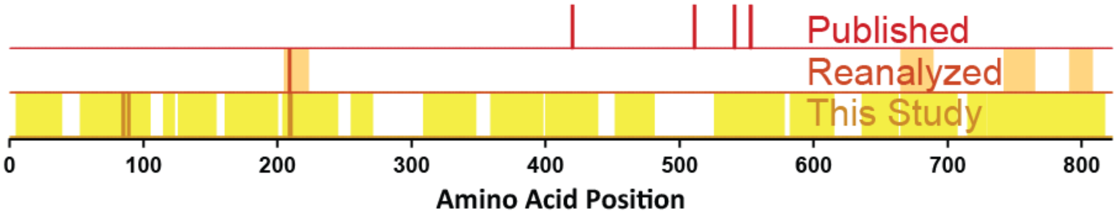

Spc98 Asynchronous

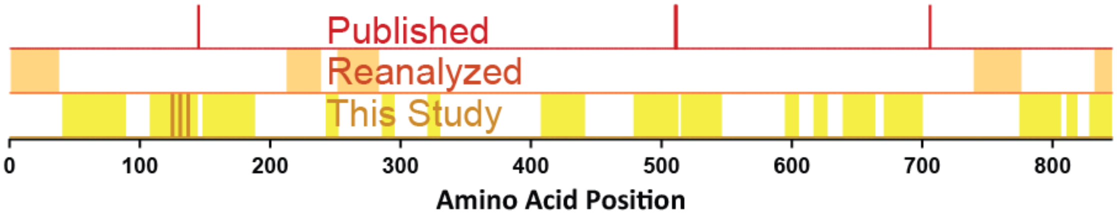

Spc98 Mitotic

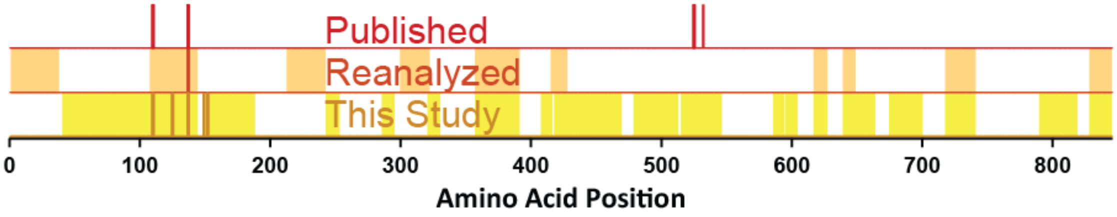

Spc98 G1

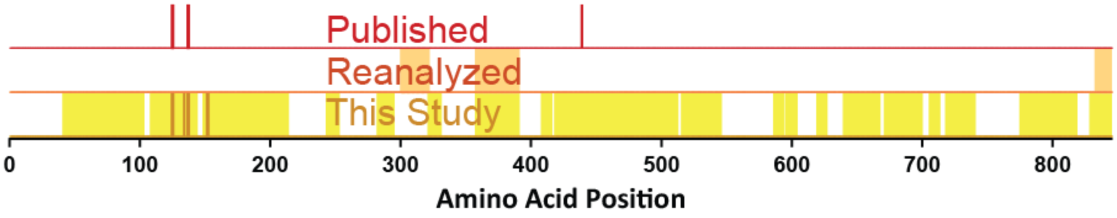

Biology Open • Supplementary information

Supplemental Figure 11. Comparison of Spc97 and Spc98 asynchronous, mitotic, and G1 phosphorylation sites. Published phosphorylation sites from Keck *et al*, 2011 are indicated as red bars. Peptide coverage of the reanalyzed Keck *et al*, 2011 data set and this data set are shown in the light orange and yellow shadings, respectively. Identified phosphorylation sites for the reanalyzed Keck *et al*, 2011 data set are indicated by the dark orange, and identified phosphorylation sites from this study are indicated by dark yellow bars. Note that the G1 data sets from Keck *et al*, 2011 were collected from SPBs isolated from cells arrested in alpha factor, while the G1 data set from this study was collected from SPBs isolated from cells arrested using a *cdc4-1* temperature sensitive mutation.

Spc110 Asynchronous

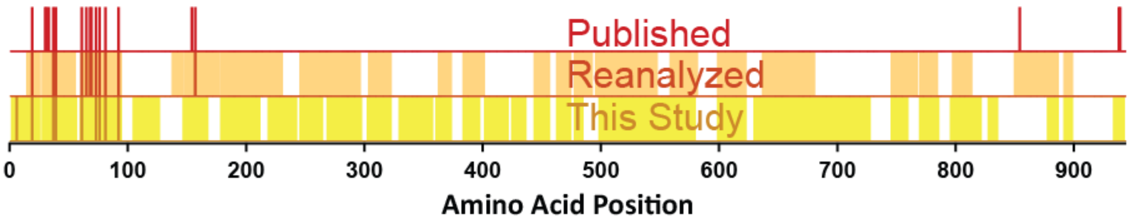

Spc110 Mitotic

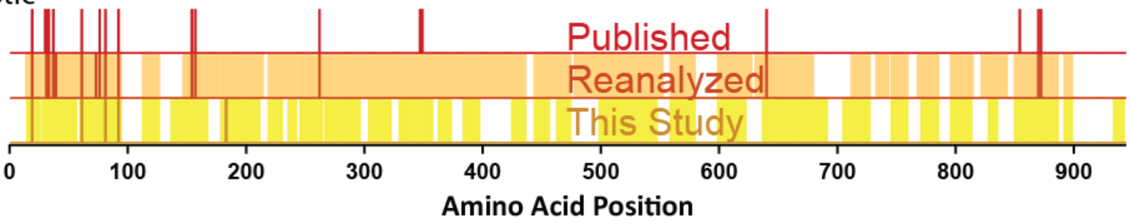

Spc110 G1

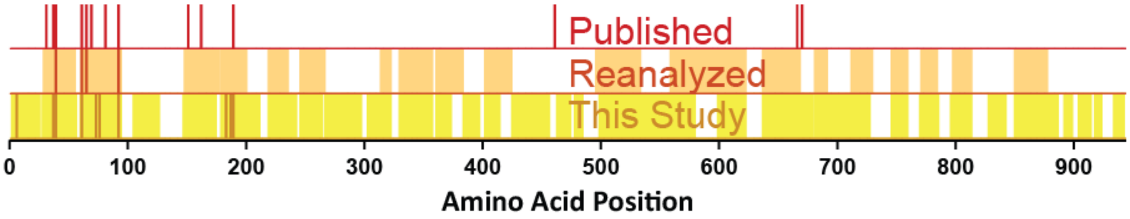

Tub4 Asynchronous

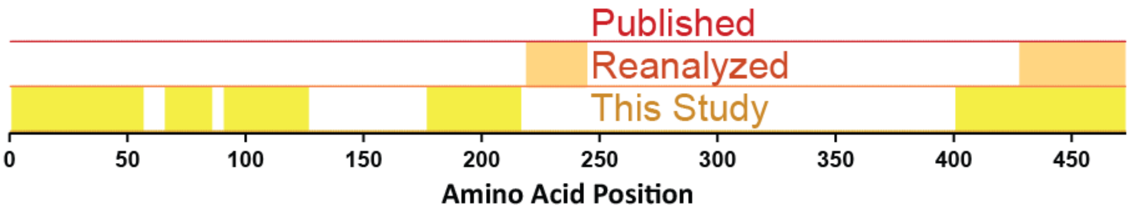

Tub4 Mitotic

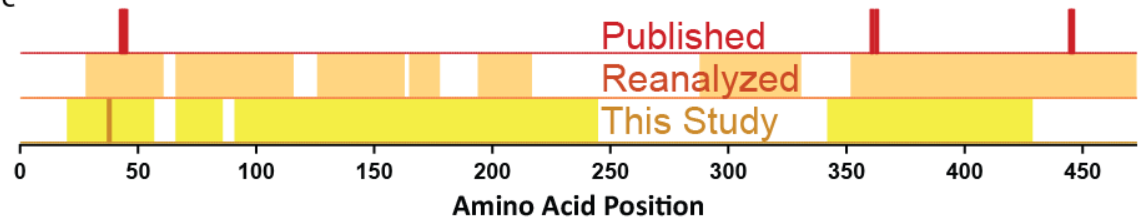

Tub4 G1

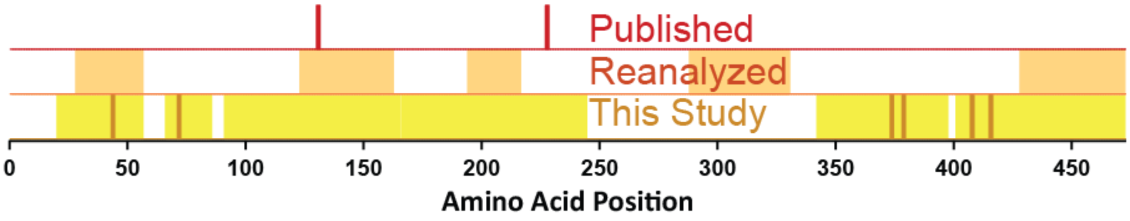

Biology Open • Supplementary information

Supplemental Figure 12. Comparison of Spc110 and Tub4 asynchronous, mitotic, and G1 phosphorylation sites. Published phosphorylation sites from Keck *et al*, 2011 are indicated as red bars. Peptide coverage of the reanalyzed Keck *et al*, 2011 data set and this data set are shown in the light orange and yellow shadings, respectively. Identified phosphorylation sites for the reanalyzed Keck *et al*, 2011 data set are indicated by the dark orange, and identified phosphorylation sites from this study are indicated by dark yellow bars. Note that the G1 data sets from Keck *et al*, 2011 were collected from SPBs isolated from cells arrested in alpha factor, while the G1 data set from this study was collected from SPBs isolated from cells arrested using a *cdc4-1* temperature sensitive mutation.

Supplementary Data S1.

Click here to Supplementary Data 1

Biology Open • Supplementary information
